# Role of Type VI secretion system in pathogenic remodeling of host gut microbiota during *Aeromonas veronii* infection

**DOI:** 10.1093/ismejo/wrae053

**Published:** 2024-03-26

**Authors:** Xiaoli Jiang, Hanzeng Li, Jiayue Ma, Hong Li, Xiang Ma, Yanqiong Tang, Juanjuan Li, Xue Chi, Yong Deng, Sheng Zeng, Zhu Liu

**Affiliations:** School of Life and Health Sciences, Hainan University, Haikou 570228, China; School of Life and Health Sciences, Hainan University, Haikou 570228, China; School of Life and Health Sciences, Hainan University, Haikou 570228, China; School of Life and Health Sciences, Hainan University, Haikou 570228, China; School of Life and Health Sciences, Hainan University, Haikou 570228, China; School of Life and Health Sciences, Hainan University, Haikou 570228, China; School of Life and Health Sciences, Hainan University, Haikou 570228, China; School of Life and Health Sciences, Hainan University, Haikou 570228, China; Wuhan National Laboratory for Optoelectronics, Huazhong University of Science and Technology, Wuhan 430074, China; Susheng Biotech (Hainan) Co., Ltd, Haikou 570228, China; School of Life and Health Sciences, Hainan University, Haikou 570228, China

**Keywords:** T6SS, bacteria competition, gut microbiome, metagenomics, host–pathogen interaction, effector-immunity protein pairs

## Abstract

Intestinal microbial disturbance is a direct cause of host disease. The bacterial Type VI secretion system (T6SS) often plays a crucial role in the fitness of pathogenic bacteria by delivering toxic effectors into target cells. However, its impact on the gut microbiota and host pathogenesis is poorly understood. To address this question, we characterized a new T6SS in the pathogenic *Aeromonas veronii* C4. First, we validated the secretion function of the core machinery of *A. veronii* C4 T6SS. Second, we found that the pathogenesis and colonization of *A. veronii* C4 is largely dependent on its T6SS. The effector secretion activity of *A. veronii* C4 T6SS not only provides an advantage in competition among bacteria *in vitro*, but also contributes to occupation of an ecological niche in the nutritionally deficient and anaerobic environment of the host intestine. Metagenomic analysis showed that the T6SS directly inhibits or eliminates symbiotic strains from the intestine, resulting in dysregulated gut microbiome homeostasis. In addition, we identified three unknown effectors, Tse1, Tse2, and Tse3, in the T6SS, which contribute to T6SS-mediated bacterial competition and pathogenesis by impairing targeted cell integrity. Our findings highlight that T6SS can remodel the host gut microbiota by intricate interplay between T6SS-mediated bacterial competition and altered host immune responses, which synergistically promote pathogenesis of *A. veronii* C4. Therefore, this newly characterized T6SS could represent a general interaction mechanism between the host and pathogen, and may offer a potential therapeutic target for controlling bacterial pathogens.

## Introduction

Gut serves as a dynamic context where bacteria engage in intricate interactions with one another and with the host, competing for scarce living resources essential for survival. Bacteria have evolved various mechanisms to compete with other species and adapt to their surroundings. These modes include mechanisms for chemical warfare via toxins (e.g. T4SS, Type VI secretion system [T6SS]), complex mechanical weapons that punch holes (e.g. Tailocins, PFT [1]), and the use of viruses in biological warfare (Phages) [2, 3]. When pathogenic bacteria invade an already-established gut micro-ecology, it will exert virulence through diverse toxic factors to remodel the microbiota, altering its composition to accommodate their needs. In the dynamic host–microbe interaction, the disruption of the original ecological balance occurs irrespective of the intentions of pathogenic bacteria, whether it be through the elimination of other bacteria or the damage inflicted on the host [4]. However, there is a lack of comprehensive study on the factors that drive this remodeling process during host–microbe interaction. Especially, the contribution of T6SS, which serves as an essential virulence mechanism, on bacterial fitness during the remodeling of host microbiota remains obscure.

T6SS is a specialized secretion system utilized by various Gram-negative bacteria, particularly those belonging to the gamma proteobacteria group, to deliver effector proteins to a diverse range of cell types, including prokaryotic and eukaryotic cells [5, 6]. It serves as a contact-dependent cell killing mechanism and has been observed in ~25% of Gram-negative bacterial species. T6SS has been documented to participate in inter-bacterial competition and pathogenesis [5, 7]. These systems are generally encoded within a large cluster of genes, and their expression is tightly regulated at multiple levels, including transcriptional, posttranscriptional, and posttranslational mechanisms [8]. This intricate control ensures the proper functioning of T6SS and the coordinated delivery of effector proteins. In *Pseudomonas aeruginosa*, the regulation of T6SS involves negative control by RsmA, a RNA binding protein in the CsrA family [9, 10]. Although the number of T6SS components varies among bacterial species, a minimum of 13 core components is required for the assembly of a functional T6SS complex, namely TssA-TssM [11]. These 13 proteins form a specialized complex that spans the bacterial envelope, resembling the tail of a contractile phage. This complex consists of a long tubular structure (Hcp/VgrG/PAAR), a spring-like sheath (TssB and TssC), and a basal plate (TssE-K) [11–13]. Upon contact with host cells or competing bacteria, the base plate of the T6SS quickly contracts the flexible sheath, propelling the long tubular structure containing the effector protein out of the cell. The tubular structure then penetrates the target cell membrane, releasing specific effector proteins that ultimately lead to death of the targeted cell [14, 15]. This dynamic mechanism enables various pathogenic bacteria to exert contact-dependent virulence on host gut epithelial cells. However, the effector mechanism is not fully delineated.

T6SS in certain bacteria is also capable of injecting effector proteins directly into host cells, leading to the disruption of cellular integrity and subsequent host cell death [9, 16–18]. T6SS can also regulate the host immune response by secreting effector proteins that interfere with host cell signaling pathways, suppress cytokine production, or inhibit the activity of host immune cells. These immune-modulating effects serve to weaken the host immune response, facilitating bacterial infection and survival [19, 20]. Traditionally, the pathogenic mechanisms attributed to T6SS-positive bacteria have focused on the direct damage inflicted on host cells. Sana *et al*. documented that *Salmonella typhimurium* utilizes a T6SS-mediated antibacterial weapon to impact symbionts directly [21]. Alternatively, taking a systems biology perspective, we propose that T6SS-mediated remodeling of the gut commensal microbiota represents an important pathogenic mechanism that facilitates bacterial niche competition and contributes to host pathogenesis.

To test this hypothesis, we choose a local prevailing pathogen strain *Aeromonas veronii* C4 that has a unique T6SS and may help to derive new information for T6SS-mediated host–pathogen interaction. Specifically, *A. veronii* C4 is a tropical isolate of *Aeromonas* as a zoonotic pathogen widely existing in water, soil, fish, and poultry with strong pathogenicity and wide drug resistance [22]. We showed that the T6SS served as a versatile weapon, specifically targeting and eliminating certain types of gut microbes, leading to the remodeling of the host microbiota. The insights gained from studying the T6SS in *A. veronii* C4 contribute to a general understanding of its mechanism of action, providing pathogenic bacteria with the ability to reshape the gut microbiome and gain adaptive advantages during host pathogenesis.

## Materials and methods

### Strains, plasmids, primers, and animals

The strains, culture conditions, and plasmids information are detailed in Table S1. Primers are listed in Table S2. Four-week-old male Kunming mice were selected and purchased from Hainan Pharmacology Research Center. All mice used in the experiment were euthanized by cervical dislocation.

#### *In vivo* colonization of *A. veronii* derivatives and pathological assessment

Mice were separately infected with 10^9^ CFU/g of *A. veronii* C4 wild type (WT), *ΔtssB*, *ΔtssB::tssB* strain, and phosphate buffer saline (PBS) control. After a 2-day infection, the kidney, cecum, and colon were dissected, weighed, and divided into two parts. One part was fixed with 4% paraformaldehyde, followed by embedding and slicing using paraffin sectioning. The sliced tissues were applied to hematoxylin and eosin staining and imaged using a Microscope (Servicebio, China). The other part was homogenized using a tissue grinder, and supernatants were plated onto Luria-Bertani (LB) agar plates containing Amp (50 μg/ml) and Kan (50 μg/ml).

### Competition assay between bacteria

Both the prey and attacker cells were adjusted to a concentration of 10^8^ CFU/ml, mixed in a 1: 1 ratio, and carefully spotted onto LB agar without antibiotics for a duration of 6 h. The recovery of prey colonies was subsequently evaluated using a colony formation assay and flow cytometry. For anaerobic *in vitro* competition experiments, all operations are meticulously carried out within the confines of the anaerobic operating station (Coy Vinyl Anaerobic Chambers, America). For *in vivo* assessment, the mammalian commensal *Escherichia coli* MG1655 was used as prey [4, 23].

### Meta gene sequencing of the mouse gut microbiome

Each group of mice, consisting of five individuals, was infected with either 10^9^ CFU/g of *A. veronii* (WT), *ΔtssB*, *ΔtssB::tssB* strain, or a PBS control. After a 2-day infection, the colon segments were dissected from the mice, rapidly frozen in liquid nitrogen, and then transported on dry ice to Biomarker (China) for subsequent analysis.

### Secretome mass spectrum analysis

The supernatants from the *ΔrsmA* (T6SS^+^), *ΔtssBΔrsmA*(T6SS^−^), and *ΔtssBΔrsmA::tssB*(T6SS^+^) were collected and sent to a mass spectrometry facility (TMT Secretome Group, Novogene, China) for analysis. Peptide information was subsequently mapped to the reference *A. veronii* standard genome (TH0426). Differential secreted proteins were analyzed by Novogene. The visualization of the differential secretome was plotted using the ggplot2 R package.

### Measurement of membrane permeability and potential

Cell membrane potential was assessed using the JC-1 kit (Beyotime, China), while cell membrane permeability was gauged through propidium iodide (PI) staining [24]. The *E. coli* BL21 strains expressing Empty, Tse1^peri^, and Tse3^peri^ were cultured overnight, and then diluted to a concentration of 10^7^ CFU/ml. These diluted cultures were transferred to a 96-well plate and subjected to growth on a shaker at 37°C for 2 h. Subsequently, Isopropyl-beta-D-thiogalactopyranoside (IPTG) was added to a final concentration of 1 mM, and the cells were grown for another 2 h. Finally, these bacteria were harvested, and their cell membrane conditions were evaluated using the JC-1 kit and PI staining.

### Cell morphology observation assay

The *E. coli* BL21 strains conferring Empty and Tse2^peri^ were induced for expression using the aforementioned method. The bacterial cultures were divided into two portions: one was stained with FM4–64 and observed using Laser Scanning Confocal Microscope (LSCM; Nikon, Japan) imaging, while the other was deposited onto a copper plate for observation of cell morphology via Transmission Electron Microscopy (TEM; Hitachi, Japan).

### Statistical analysis

The statistical analysis methods included the one-way analysis of variance (ANOVA) test, *t*-tests, and the Kruskal–Wallis rank sum test. Statistical details (sample size, test values, *P* values) for each experiment were provided in the respective figure legends. The RStudio package (ggtree) was utilized for drawing the evolutionary tree. Statistical analysis of results was conducted with GraphPad Prism version 8.3 (GraphPad software Inc.; San Diego, CA), using *P* value of <.05 as statistically significant.

Additional methods are described in Supplementary materials and methods.

## Results

### *A. veronii* C4 encodes a functional T6SS cluster

**Figure 1:**
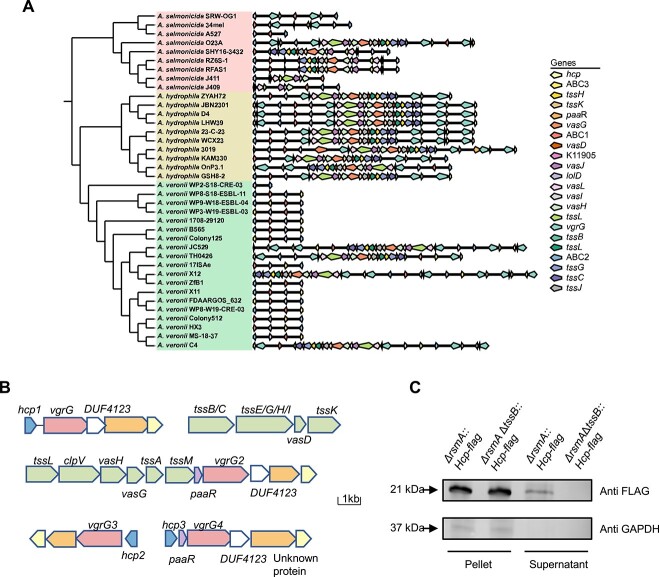
*A. veronii* C4 encodes a functional T6SS cluster; (A) phylogenetic tree and comparative analysis of structures of T6SS in varied strains; (B) schematic illustration depicting the T6SS gene clusters in *A. veronii* C4; (C) western blot analysis of HCP secretion in the indicated strains, including flag-tagged HCP expressed in *ΔrsmA* or *ΔrsmAΔtssB*; HCP was detected using an antiflag antibody in pelleted cells or supernatants; the housekeeping non-secreted protein GAPDH served as a loading control; for complete western blot data, see Fig. S1.

Our comparative genomic analysis of *Aeromonas salmonicida*, *Aeromonas hydrophila*, and *A. veronii* strains showed that the T6SS gene cluster was significantly different among different species in the same genus. Although all 10 strains of *A. hydrophila* and 4 out of 9 strains of *A. salmonicida* contained a complete T6SS system, only 4 out of 20 strains of *A. veronii* possessed a complete T6SS. Among *A. veronii*, strains JC529, TH0426, and X12 were found to be closely related, whereas *A. veronii* C4 strain revealed a distinct separation from other species (Fig. 1A) and identification of a functional T6SS (Fig. 1B). Therefore, a comprehensive characterization of the T6SS in *A. veronii* C4 is needed to gain valuable insights into the uncanonical mechanisms through which T6SS contributes to bacterial competition and virulence.

To validate the function of the T6SS in *A. veronii* C4, we created a T6SS reporter strain (Δ*rsmA::HCP*) by deleting the expression of the T6SS transcriptional suppressor *rsmA* [9] and introducing ectopic expression of flag-tagged hemolysin co-regulation protein (HCP), a bona fide substrate of T6SS. Previous studies have shown that T6SS can be activated through the knockout of the RNA-binding protein RsmA in a cell autonomous manner in *P. aeruginosa* [25]. Utilizing *ΔrsmA* as the parental strain, it was ascertained that within the cytoplasm of both the T6SS-activated strain *ΔrsmA* and the T6SS-deficient strain *ΔtssBΔrsmA*, the HCP-flag protein was regularly expressed at comparable level (Fig. 1C). However, a distinct disparity emerged in the culture supernatant, where the T6SS-deficient strain *ΔtssBΔrsmA* exhibited an incapacity to secrete the HCP protein, rendering it undetectable under these conditions. In stark contrast, the culture supernatant of the T6SS-activated strain *ΔrsmA* exhibited unimpeded secretion of the HCP protein (Fig. 1C). These findings indicated the presence of a functional T6SS in *A. veronii* C4, and the deletion of the structural component *tssB* significantly impaired the substrate secretion activity of the *A. veronii* T6SS.

### T6SS promotes ecological niche colonization and host pathogenesis

**Figure 2:**
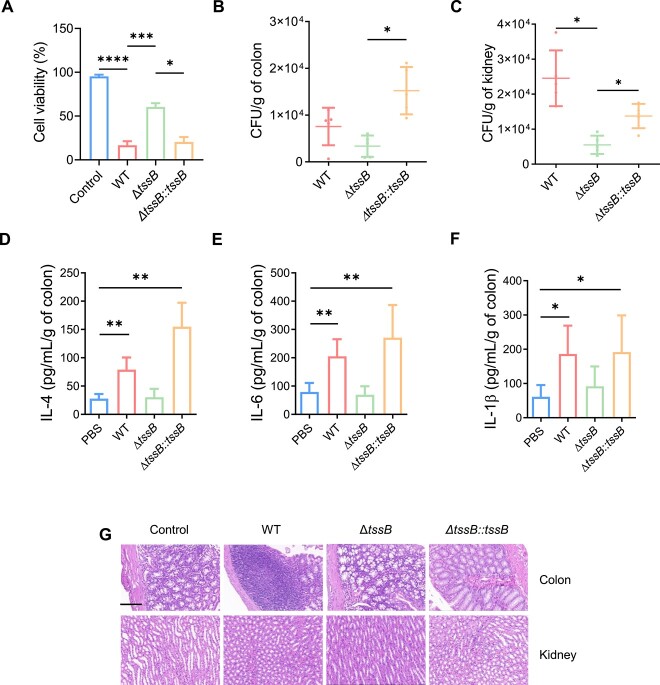
T6SS promotes ecological niche colonization and host pathogenesis; (A) cell viability analysis of mouse macrophage cell line infected with *A. veronii* derivatives; the three variants were WT, *ΔtssB*, and *ΔtssB::tssB* strain; a control group without bacterial infection was included (*n* = 3); (B and C) quantification of colony forming units (CFU) of WT, *ΔtssB*, and *ΔtssB::tssB* strain isolated from three tissues of infected mice including colon (B) and kidney (C); (D–F) assessment of cytokine levels in the colon of mice infected with WT, *ΔtssB*, *ΔtssB::tssB*, or saline control (PBS); data in panels A–F were shown as mean ± SD, with error bars indicated the standard deviation; statistical significance was determined by one-way ANOVA test, *P* < .0001(^****^), *P* < .0002(^***^), *P* < .01(^**^), 0.01 < *P* < .05(^*^), “ns” indicated not significantly different; *n* = 5 for B–F; (G) histological examination (HE) of pathological alteration in the colon and kidney of mice infected with WT, *ΔtssB*, and *ΔtssB::tssB* strains of *A. veronii*; *n* = 5 for each experimental group; scale bars were shown with indicated length (50 μm).

Studies conducted on other bacteria have demonstrated the crucial role of T6SS in bacterial pathogenesis [13, 26, 27]. To explore whether the T6SS in *A. veronii* C4 also contributed to its pathogenicity, the cultured mouse macrophages *in vitro* were infected with either the WT or *ΔtssB* mutant strain of *A. veronii*, and their cellular toxicity was compared (Fig. 2A). The results revealed a significant reduction in toxicity when *tssB* was deleted, indicating the requirement of T6SS for *A. veronii* pathogenesis. To further confirm the contribution of *tssB* to T6SS-mediated *A. veronii* virulence, we found that the defect of cell toxicity in the *ΔtssB* mutant was fully restored by expressing *tssB* (Fig. 2A).

The T6SSs of various enteric pathogens, such as *S. typhimurium* and *Vibrio cholerae*, have been proposed to facilitate colonization and induce disease through the selective targeting of microbial competitors [9, 28]. Consistent with this, based on the optimal infection time as determined (Fig. S2), we observed that the colonization of *A. veronii* C4 significantly decreased in the mouse colon and cecum when the T6SS was absent compared to the complemented strains (*ΔtssB::tssB*). However, compared with WT, it only tantalizingly decreased but did not reach significant difference. This may be due to variation between mice and the large intra-group error caused by cage effects (Fig. 2B and Fig. S3A). We observed the same phenomenon in the mouse kidneys as well (Fig. 2C). It is postulated that *A. veronii* may manifest a robust infectivity toward the renal system. Moreover, T6SS-sufficient *A. veronii* elicited stronger immune responses within the primary infection site, the colon of the host mice, compared to the *ΔtssB* mutant. *A. veronii* infection stimulated an elevation of inflammatory cytokines IL4, IL6, and IL1β, indicating that T6SS was one of the local immunogenic components of *A. veronii* (Fig. 2D–F). Meanwhile, we discovered that the mice's body immunity was activated as long as they were gavaged *A. veronii*, regardless of whether the *A. veronii* contained T6SS (Fig. S3B and C). Pathological staining of kidney and colon slices showed that infection with WT and *ΔtssB::tssB*, but not *ΔtssB* mutant, resulted in substantial damage to the kidney and ulcer-like necrosis in the colon (Fig. 2G). In contrast to colon necrosis, the cecum showed only edema in the T6SS sufficient group (Fig. S3D). Taken together, these findings demonstrated that the T6SS was required for the host colonization and pathogenesis of *A. veronii* C4.

### T6SS mediates interbacterial competition *in vivo* and *in vitro*

**Figure 3:**
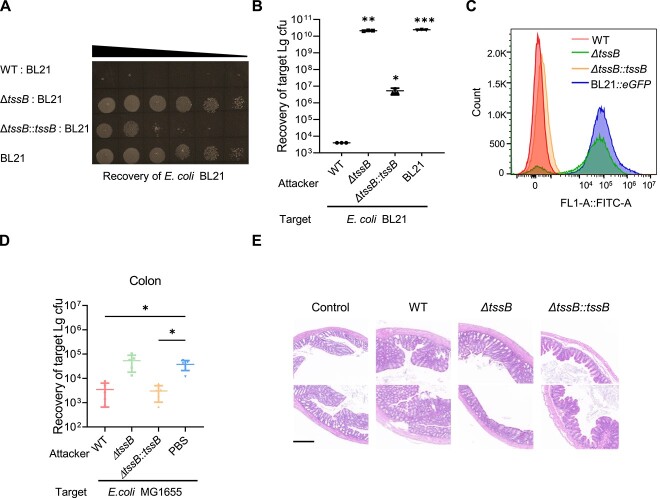
T6SS mediates interbacterial competition *in vivo* and *in vitro*; (A–C) the growth inhibition of *A. veronii* on *E. coli* BL21 mediated by functional T6SS; (A) representative images of BL21 recovery assay after co-incubation with serial dilutions of *A. veronii* variants, including WT, *ΔtssB*, *ΔtssB::tssB*, and *E. coli* BL21 only; (B) quantification of surviving rates of *E. coli* BL21 after co-incubation with aforementioned strains (*n* = 3); (C) flow cytometry analysis was performed to evaluate the survival of *E. coli* BL21, which indicated by eGFP expression, after co-incubation with different variants of *A. veronii*, by using the FITC channel; (D) survival rates of gut commensal *E. coli* MG1655 isolated from mouse colon after *in vivo* colonization with WT, *ΔtssB*, and *ΔtssB::tssB*, as assessed by colony forming units; (E) morphology of host colon after infection of saline (control), WT, *ΔtssB*, and *ΔtssB::tssB*; scale bars were shown with indicated length (100 μm); data in panels B–D were shown as mean ± SD, with error bars indicated the standard deviation; statistical significance was determined by one-way ANOVA test: *P* < .0002(^***^), *P* < .01(^**^), and 0.01 < *P* < .05(^*^) denoted significance, while “ns” indicated not significant difference; *n* = 5 for panel D.

To elucidate the physiological role of the newly identified T6SS in *A. veronii* C4, we assessed the inter-bacterial competition capacity of strains with intact and deficient *tssB* genes. We referred to Anderson’s approach to bacterial competition [4]. To this end, *E. coli* BL21 were co-cultured with various derivatives of *A. veronii* C4, including WT, *ΔtssB*, and *ΔtssB::tssB*. The growth of *E. coli* BL21 was significantly suppressed by the WT, whereas this inhibitory effect was greatly attenuated in the *ΔtssB* mutant (Fig. 3A and B). Moreover, the inhibitory effect of *A. veronii* C4 *ΔtssB* toward *E. coli* BL21 growth was restored when *tssB* was expressed (Fig. 3A and B). In addition, we devised a flow cytometry procedure to quantitatively evaluate the competition between *A. veronii* C4 and genetically labeled fluorescent *E. coli* BL21*::eGFP*. Consistently, the co-culture with *A. veronii* C4 WT strain resulted in pronounced suppression of *E. coli* BL21 growth compared to *E. coli* BL21 alone (Fig. 3C). These results of bacterial competition between *A. veronii* C4 and *E. coli* DH5α and *E. coli* MG1655 were also consistent (Fig. S4A–F). Intra-species competition between WT and the mutants deficient in T6SS *(ΔtssB* or *ΔsmpB* [29]) of *A. veronii* C4 was assessed in a similar experimental setting (Fig. S4G and H). WT strain did not have a competitive advantage against its mutants, as bacteria have evolved homologous immune proteins to counteract the toxic effects of the effector proteins and prevent self-poisoning. The above results indicated that the T6SS conferred inter-bacterial competitive advantage to *A. veronii C4*, but the presence of homologous immune proteins did not affect the growth of sister strains.

To confirm if T6SS conferred competitive advantages under physiological context, we examined the toxicity of T6SS-sufficient and T6SS-deficient *A. veronii* C4 on the established gut microbiota. To reduce the complexity of the intestinal ecosystem, the guts of the host mice were treated with a combination of antibiotics and precolonized with the gut commensal *E. coli* MG1655. Subsequently, the mice were intragastrically administered by either the WT *A. veronii* C4, *ΔtssB*, or *ΔtssB::tssB* strains. *In vivo* competition experiment, the survival numbers of *A. veronii* C4 and MG1655 in feces were counted over time (Figs S5A–F and S6A–F). The results showed that the recovery rate of *A. veronii* C4 coculturing with *E. coli* MG1655 is higher than that of *A. veronii* growing alone albeit only on the second day of infection (Fig. S5B). This indicated that killing of the prey strain can slightly benefit *A. veronii* C4, because there is no significant difference at other time points. With the increase of infection time, the amount of MG1655 showed a significant decrease after competition with T6SS sufficient *A. veronii* C4 (Fig. S6A–D). The colony forming units of MG1655 isolated from the colon were significantly reduced by the WT and *ΔtssB::tssB*, but not by the *ΔtssB* mutant (Fig. 3D). Consistent with the T6SS-mediated toxicity toward eukaryotic host cells, we observed swelling and ruptures even necrosis of intestinal epithelial cells in colon tissues infected with the WT and *ΔtssB::tssB*, while no obvious damage was found in the *ΔtssB* mutant, as revealed by pathological staining (Fig. 3E). These findings supported the physiological role of T6SS in combating both competing gut microbiota and promoting pathogenesis during host infection.

### T6SS reshapes host gut microbiota during *A. veronii* infection

**Figure 4:**
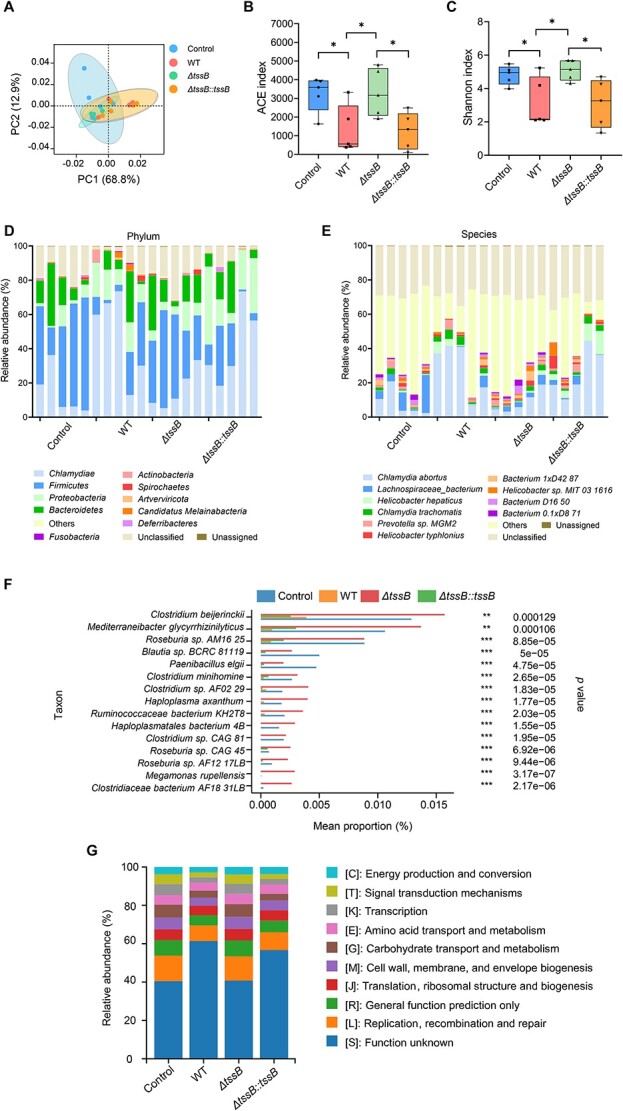
T6SS reshapes host gut microbiota during *A. veronii* infection; (A) PCA was used to analyze the data, visually represented by colored ellipses and points; (B and C) ACE index and Shannon index are used to showcase alpha diversity; statistical significance was determined by *t*-test: 0.01 < *P* < .05(^*^) denoted significance, while “ns” indicated not significant difference; (D and E) phylum and species composition distribution: the *x*-axis represented sample names, and the *y*-axis represented relative abundance percentage; (F) the differences in species distribution among groups; statistical significance was determined by Kruskal–Wallis rank-sum test: *P* ≤ .0001(^***^), *P* ≤ .001 (^**^); (G) functional differences, eggNOG analysis.

To investigate the role of T6SS in *A. veronii* infection, we examined its impact on the composition of commensal gut microbiota under normal conditions. Principal component analysis (PCA) revealed a pronounced shift in gut microbiota composition in mice infected with WT compared with saline as negative control, but not in those infected with the T6SS-deficient *ΔtssB* mutant, suggesting a major impact of T6SS on host microbiota remodeling (Fig. 4A and Fig. S7A and B). However, we found a relatively large intra-group variation. This might be due to difference in host immune status and cage effects.

Alpha diversity of the gut microbiota was markedly reduced in WT infection but not in *ΔtssB* infection, and the complementation of *tssB* in the *ΔtssB* mutant (*ΔtssB::tssB*) restored the impairment of gut microbiota diversity (Fig. 4B and C and Fig. S7C and D). The relative abundance of different microbes at various taxonomic levels (phylum, class, order, family, genus, and species) was significantly altered during WT infection (Fig. 4D and E and Fig. S7E–H), which was similar to that of mice infected with *ΔtssB::tssB*, the mice infected with *ΔtssB* was not significantly different from that in the saline control mice. Thus, the diversity of the gut microbiota was highly correlated with the absence of T6SS in infecting *A. veronii*.

The most prominently decreased gut microbiota species caused by *A. veronii* infection included *Lachnospiraceae bacterium*, *Clostridium beijerinckii*, *Mediterraneibacter-glycyrrhizinilyticus*, *Roseburia*, *Blautia*, and *Megamonas* (Fig. 4E and F), all of which are known as beneficial anaerobic bacteria colonizing the gut [30–39]. *In vitro* competition assay, the T6SS sufficient strain *A. veronii* C4 (T6SS^+^) significantly inhibited the growth of *L. bacterium*. On the contrary, this inhibitory effect on *L. bacterium* was significantly attenuated by T6SS deficiency in *A. veronii* C4 *ΔtssB*. This result suggested that T6SS of *A. veronii* C4 confer a competitive advantage over a representative commensal gut bacteria *in vitro* (Fig. S8A and B). In particular, strains such as *L. bacterium*, *C. beijerinckii*, *Roseburia*, *Megamonas*, and *Blautia* are associated with butyrate production, which is particularly important for maintaining homeostasis of the intestinal epithelium cells [31, 37, 40–43]. Therefore, we speculate that the reduction of these beneficial bacteria is the underlying cause of the pathogenicity of *A. veronii* toward the host. We found an increase of *Helicobacter*, *Chlamydia abortus*, *Chlamydia trachomatis*, and *Retrovirus* following WT or *ΔtssB::tssB* infection, but not in the T6SS-deficient *ΔtssB* infection (Fig. 4D and E). These findings suggested that *A. veronii* infection disrupted the balance of the host gut microbiota, potentially leading to concurrent infections by other microbes. Thus, T6SS appeared to negatively impact the diversity of the gut microbiota during *A. veronii* infection.

We also found a significant decrease in the total number of genes in the gut microbiota of mice infected with *A. veronii* WT or *ΔtssB::tssB*, while there was no significant difference between the *ΔtssB* and control groups (Fig. S9A and B). To further investigate the variations in the gut microbiota environment across different treatment groups, we conducted eggNOG analyze (Fig. 4G). The results revealed significant downregulation of pathways related to energy metabolism, signal transduction, gene transcription, amino acid and carbohydrate transport and metabolism, cell wall synthesis, and DNA replication, recombination, and repair in the WT and *ΔtssB::tssB* groups. However, no significant differences were observed in these metabolic pathways between the *ΔtssB* group and the control group. Further analysis from CAZy enzyme activity examination unveiled a substantial decrease in the expression of specific enzymes associated with carbohydrate metabolism in the WT and *ΔtssB::tssB* groups (Fig. S9C). Carbohydrates play a crucial role in shaping the microbial community [44]. Mammals lack many enzymes necessary for carbohydrate degradation in their gut, thus relying on resident gut bacteria to fulfill this enzymatic requirement [45]. Gut bacteria degrade these organic substances to meet their own survival demands while producing short-chain fatty acids as a feedback mechanism to the host [46, 47]. Short-chain fatty acids possess antiinflammatory properties and can regulate epigenetic remodeling and influence host metabolism [47], such as butyrate production [31]. The interaction among carbohydrates, the microbial community, and host health predominantly occurs in a stable manner. Moreover, the analysis conducted using virulence factor database (VFDB), revealed a noteworthy downregulation of genes associated with virulence factors, including VF0268, VF0286, and VF0272 (Fig. S9D). The downregulation of these essential genes involved in metal ion acquisition and growth (Table S3) in both the WT and *ΔtssB::tssB* groups resulted in a decrease in gut bacteria diversity. Additionally, consistent results were observed for genes related to antibiotic resistance, with significant downregulation observed in the WT and *ΔtssB::tssB* groups (Fig. S9E). Conversely, no significant expression difference of these genes were observed between the *ΔtssB* group and the control group (Fig. S9E). In summary, the *A. veronii* T6SS actively reshapes the gut microbiota environment to promote its own proliferation at the expense of commensal bacterial strains, thereby augmenting its pathogenicity toward the host.

### Identification of T6SS secreted proteins through secretomic analysis

**Figure 5:**
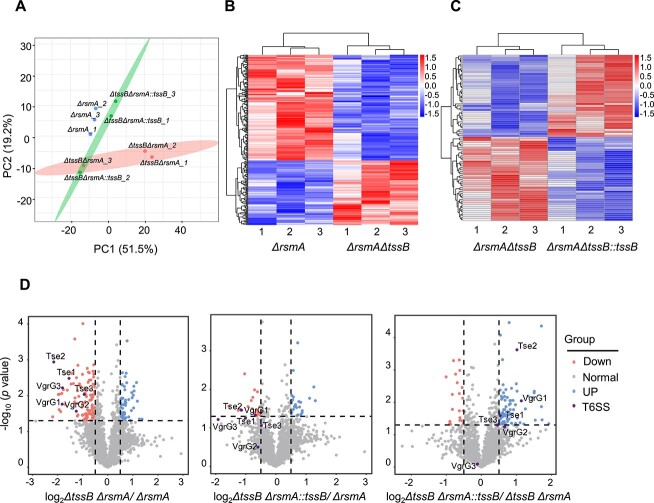
Identification of T6SS secreted proteins through secretome analysis; (A) PCA of secreted proteins from *ΔrsmA*, *ΔrsmAΔtssB*, and *ΔrsmAΔtssB::tssB* (*n* = 3); (B, C) heatmap showed clusters of differentially secreted proteins among the three strains mentioned in panel A; (D) volcano plots depicting indicated comparisons; the cutoff for *P* value was set to .05, while the cutoff for fold changes was set to 0.5.

Our characterization of the newly discovered T6SS in *A. veronii* has provided evidence suggesting its positive role in gaining evolutionary fitness, likely through the release of toxic effectors that are harmful to both the host and competing gut microbiota. However, the downstream effector substrates specific to the *A. veronii* T6SS remain largely unknown. To gain unbiased insights into the downstream toxicity mechanism of the T6SS, we performed secretomic analysis using *ΔrsmA*(T6SS^+^), *ΔtssBΔrsmA*(T6SS^−^), and *ΔtssBΔrsmA::tssB*(T6SS^+^). PCA results showed that there was a small intergroup data difference between *ΔrsmA*(T6SS^+^) and *ΔtssBΔrsmA::tssB* (T6SS^+^), while both groups exhibited significant intergroup differences compared to *ΔtssBΔrsmA*(T6SS^−^) (Fig. 5A). Notably, *tssB* knockout resulted in a decrease in the secretion of certain types of proteins (downregulated DEGs: 154), while also leading to an increase in the secretion of other proteins (upregulated DEGs: 95). Furthermore, ectopic expression of *tssB* did not fully restore the differential secretion pattern in the T6SS-deficient *A. veronii*, suggesting the existence of a dynamic feedback regulatory mechanism to compensate for T6SS loss (Fig. 5B and C). However, there were more downregulated proteins than upregulated ones in the *tssB* knockout. Many of the T6SS components encoded by the T6SS gene clusters fell into the downregulated category. These proteins included well-documented T6SS subunits HCP, PAAR repeat-containing protein (PAAR), Valine-glycine repeat protein G (VgrGs), as well as Tse1, Tse2, and Tse3, whose functions were not clear (Fig. 5D). The secretion of bona fide HCP1–3 proteins, VgrG1–4, PAAR1, and PAAR2 was also dampened in the absence of *tssB* and restored upon overexpression of *tssB* in the *ΔtssB* mutant (Fig. S10A and B).

### Functional validation of intracellular and extracellular effector proteins in *A. veronii* C4

**Figure 6:**
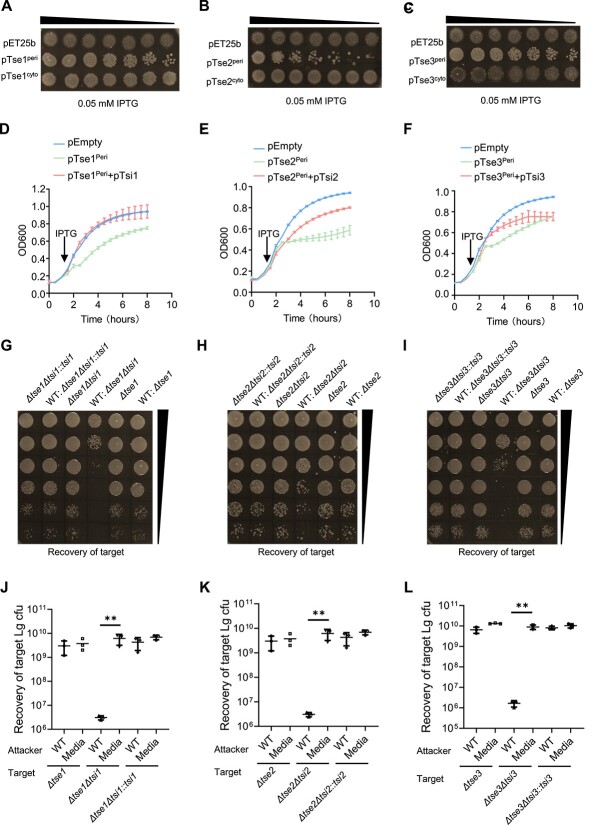
Functional validation of intracellular and extracellular effector proteins in *A. veronii* C4; (A–C) intra-species cell toxicity of indicated effector proteins that were inducible expressed by IPTG in secreted (peripheral expression, peri) form or cytosolic (cyto) form; (D–F) growth inhibition assay of effector proteins Tse1, Tse2, or Tse3, with or without co-expression with their cognate immunity proteins, Tsi1, Tsi2, and Tsi3; the arrows in the figure represented the time points for adding IPTG; (G–I) intra-species competition experiments were conducted to validate whether these effector-immunity (EI) pairs were functional in *A. veronii*; (J–L) quantification of surviving rates of target strain after co-incubation with *A. veronii* WT; statistical significance was determined by one-way ANOVA test: *P* < .01(^**^) indicated significance; *n* = 3 for each group.

Secretomic analysis has identified several differentially expressed genes dependent on the T6SS in the conditional medium of *A. veronii* C4 culture. Among these genes, there were several encoded proteins from the T6SS gene locus, including Tse1, Tse2, and Tse3, which have not been well characterized (Fig. S11A). Comparative analysis of amino acid sequences indicates that Tse1 and Tse3 have a homology of up to 60.3%. Both of them contain a conserved domain, DUF2235, which is of unknown function. Tse2 has a high amino acid sequence homology with an unknown protein of *Proteobacteria* strain, and it contains an N-terminal peptidase M23 and a C-terminal Lyz-like domain. Based on their predicted structural components of the T6SS and the presence of a DUF2235 domain or a Lyz -like domain [48–53], we hypothesized that these proteins may have effector activity (Fig. S11B and C). To test this hypothesis, we expressed these proteins targeted to different subcellular locations, namely the pericellular space or cytosol. Expression of pericellularly targeted Tse1, Tse2, or Tse3 exhibited toxic effects and inhibited the growth of co-cultured *E. coli* BL21, while the growth of *E. coli* BL21 was unaffected when the expression of these three proteins was confined to the cytoplasm (Fig. 6A–C). Thus, Tse1, Tse2, and Tse3 were likely effector proteins that were secreted by the T6SS in *A. veronii*. Since genes Tsi1, Tsi2, and Tsi3 were encoded within the same operon and arranged adjacent to Tse1, Tse2, and Tse3, respectively, we suspected that these proteins could have counter-acting activity toward their corresponding effector proteins. Therefore, we expressed protein pairs of Tse1-Tsi1, Tse2-Tsi2, or Tse3-Tsi3 with a pericellular targeting signal peptide. The toxicity of pericellularly targeted Tse1 protein appeared to be neutralized by Tsi1 (Fig. 6D). The similar inhibitory effect was observed for Tse2 and Tsi2, Tse3, and Tsi3 proteins (Fig. 6E and F). Distinguished from previously reported head-to-end tandem expressions of the effector and the corresponding immune protein, *tsi2* gene and *tse2* gene exhibited a distinctive genomic localization, forming a chimera characterized by a 29-base pair overlap (Fig. S11D). Moreover, this finding implied that bacteria employed their constrained genome capacity to optimize their functional efficiency.

To further validate the functions of these proteins within *A. veronii*, we constructed mutant strains *Δtse1*, *Δtse2*, and *Δtse3*, which were then competed against the WT strain. We found that WT strain was unable to kill the mutant strains. However, when the corresponding homologous immunity proteins *Δtse1Δtsi1*, *Δtse2Δtsi2*, and *Δtse3Δtsi3* were simultaneously mutated in the mutant strains, their growth was significantly inhibited by WT strain (Fig. 6G–I). Furthermore, the growth inhibition was disappeared when the respective immunity protein was complemented. In summary, we have identified Tse1, Tse2, and Tse3 as toxic effector proteins secreted by the T6SS of *A. veronii*, while Tsi1, Tsi2, and Tsi3 are their respective homologous immunity proteins.

### T6SS-dependent effector proteins selectively target the cell membrane or cell wall in *A. veronii*

**Figure 7:**
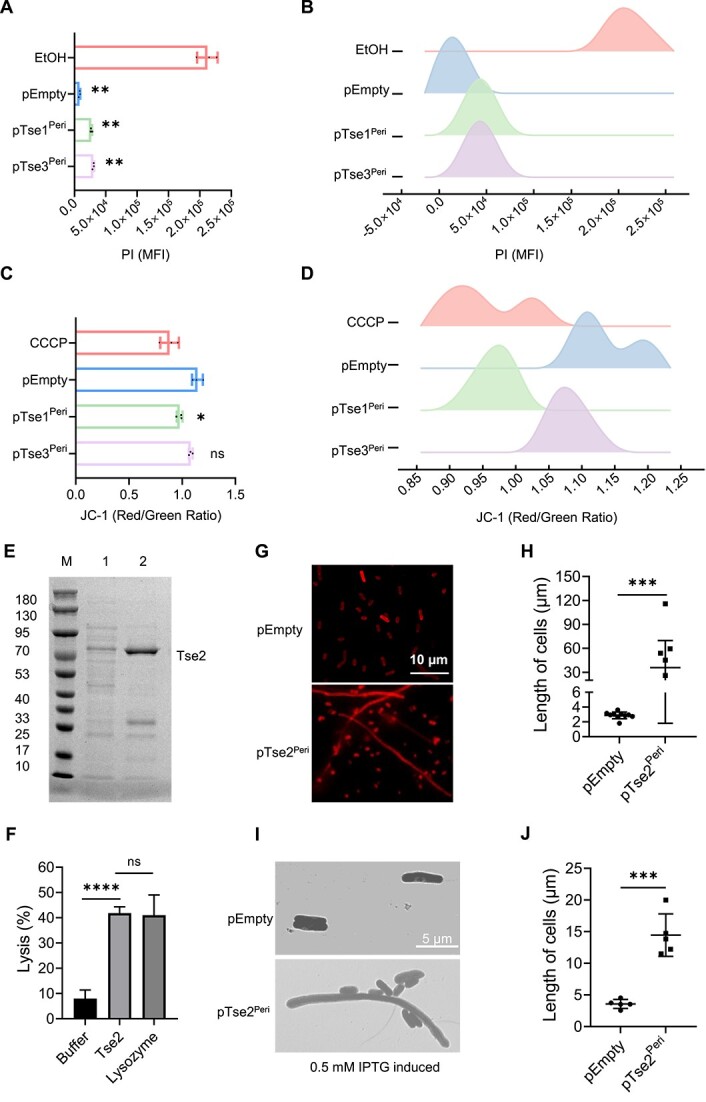
T6SS-dependent effector proteins selectively target the cell membrane or cell wall in *A. veronii*; (A and B) PI staining of *A. veronii* treated with ethanol (EtOH), or transformed with variants plasmids, including empty vector (pEmpty), plasmid expressing secreted form of Tse3 (pTse3^Peri^), or secreted form of Tse1 (pTse1^Peri^); the flow cytometry analysis of PI-stained *A. veronii* was shown on the right; (C and D) JC-1 staining of *A. veronii* treated with oxidative phosphorylation chain inhibitor CCCP, or ectopically expressing secreted forms of effector proteins Tse3, Tse1, or empty vector; the flow cytometry analysis of CCCP stained *A. veronii* was shown on the right; mean fluorescence intensity (MFI) in panels A and C was shown as mean ± SD; (E) Tse2 was purified and analyzed by SDS-PAGE gel electrophoresis; (F) cell lysis rate data statistics; statistical significance was determined using one-way ANOVA test: *P* < .0001(^****^) indicated significance, while “ns” represented no significant difference; *n* = 3 for each group; (G and H) LSCM imaging of bacterial morphology; scale bars were shown with indicated length 10 μm; *n* = 10 for each group; (I and J) TEM imaging of bacterial morphology; scale bars were shown with indicated length 5 μm; statistical significance was determined using *t-*test: *P* < .0002(^***^), *P* < .01(^**^), and .01 < *P* < .05(^*^) indicated significance; *n* = 5 for each group.

Based on the structural analysis and functional validation of the aforementioned effector proteins, it has been discovered that both Tse1 and Tse2 harbor a conserved DUF2235 domain, which incorporates a G-X-S-G lipase motif (Fig. S11B). Additionally, these proteins exhibit localization in the periplasmic compartment, where they exert their functional activities. Drawing upon these observations, we hypothesize that these two proteins hold the capacity to selectively target the cell membrane by perturbing its structural integrity. Membrane disruption should result in dissipation of the membrane potential and in ion leakage. Therefore, we investigated the influence of periplasmic Tse1 and Tse3 effectors on the membrane potential and permeability of *E. coli*, following the methods outlined in the published article [24]. The presence of secreted Tse1 and Tse3 proteins compromised the permeability of the cell membrane, albeit to a lesser extent than the positive control (ethanol treatment) (Fig. 7A and B). Periplasmic expression of Tse1 and Tse3 proteins compromised the membrane potential, with Tse1 overexpression nearly eliminating it to a level comparable to treatment with CCCP, a membrane depolarizing reagent (Fig. 7C and D). These evidences supported the notion that effector proteins Tse1 and Tse3 executed cell toxicity by damaging plasma membranes of the target cells.

The bioinformatic structural analysis of the effector protein Tse2 mentioned above suggested that it contained a peptidase M23 domain at the N-terminus and a Lyz-like domain at the C-terminus (Fig. S11C). We were aware that M23 plays a crucial role as a peptidoglycan hydrolase in bacterial growth and division, whereas Lyz-like constitutes a lysozyme-like structural domain associated with cell wall lysis [53–55]. To delve deeper into the cytotoxic mechanism triggered by Tse2, we designed *in vitro* and *in vivo* biochemical experiments. *In vitro* biochemical experiments, recombinant Tse2 was expressed and purified using pET28a system in *E. coli* BL21 (plysS). Given that Tse2 exhibits lysozyme-like activity in lysing cell walls, we used lysozyme as a positive control and PBS as a negative control to conduct an *in vitro* lysis experiment on polymyxin B-treated *E. coli* BL21 cells (Fig. 7E and F). The ultimate findings demonstrated that upon exogenous addition to the cell culture, the purified Tse2 protein exhibited cell lysis capabilities akin to lysozyme. For the *in vivo* biochemical experiment, we used TEM and LSCM were employed to visualize the morphological characteristics of control bacteria and Tse2-expressing bacteria within the periplasmic space (Fig. 7G and H). The findings demonstrated that Tse2 expression in the periplasm led to anomalous bacterial division, resulting in the formation of elongated spindle-shaped cells that were 2–10 times longer in diameter than normal cells (Fig. 7H and J). On the grounds of these observations, we postulated that the effector protein Tse2 disrupts the integrity of the bacterial cell wall, thereby impeding normal bacterial division.

Based on the aforementioned results of *in vitro* inter-species competition (Fig. 3A and B), we aimed to elucidate the specific T6SS-secreted effector protein accountable for the observed effects. To address this, competitive experiments were meticulously designed, pitting *A. veronii* mutant strains *Δtse1*, *Δtse2*, and *Δtse3* against *E. coli* BL21 (Fig. S12A and B). The outcomes unequivocally demonstrated a pronounced decrement in the growth inhibition of *E. coli* BL21 solely in the absence of the effector protein Tse2, whereas the deficiency in Tse1 and Tse3 did not attenuate the growth inhibition against BL21. Consequently, we assert that the effector protein Tse2 exerts a paramount influence in the interspecies competition involving *A. veronii* and *E. coli* BL21.

## Discussion

Globally, over 3 million people die each year from diarrhea caused by gut pathogenic bacteria infections [56, 57]. Gut pathogenic bacteria utilize multiple strategies, including T6SS, to exert their virulence. However, the molecular mechanism by which T6SS promotes pathogenesis during bacterial infections remains unclear. The elucidation of the molecular mechanism of T6SS is so far mainly based on *in vitro* experiments, such as competition experiments with laboratory-cultured *E. coli* strains. The role of T6SS in the context of physiological situation, particularly within host-pathogen symbiotic systems, remains understudied.

*A. veronii* poses a severe threat to the health of human being and farm animals, but the exact mechanism by which it interacts with the host and gut microbiota is still unknown. Here, we sequenced the genome of pathogenic *A. veronii* C4 strain and characterized a species-specific T6SS. Our findings provided evidence that this newly identified T6SS functions as a secretion machinery, releasing effector proteins like HCP. We also demonstrated the essential role of the T6SS in the survival and pathogenesis of *A. veronii* C4 by outcompeting gut commensal microbiota. Additionally, we observed *A. veronii* infection significantly impaired the diversity of the host mouse gut microbiota. Lastly, employing a secretomic approach, we identified novel T6SS dependent effector proteins and neutralization proteins.

The gut represents a dynamic physiologically complex environment. The commensal microbiota plays a crucial role in limiting *A. veronii* infection by occupying niche spaces and competing for limited resources necessary for the survival of the bacteria. Conversely, *A. veronii* must overcome established gut commensal bacteria and evade surveillance by resident immune cells. Thus, the evolutionary pressure on *A. veronii* necessitates the delivery of multiple effectors by T6SS to the extracellular matrix. These effectors not only restrict the growth of gut microbiota but also kill eukaryotic cell. Through metagenomic sequencing analysis of the gut microbiota, we observed changes in the distribution and diversity of intestinal species in mice infected with *A. veronii* carrying a functional T6SS (Fig. 4A–E). The diversity of gut species significantly decreased, coupled with a notable reduction in their relative abundance, especially among specific beneficial bacteria associated with antiinflammatory properties in the intestines, which exhibited a substantial decrease or even disappearance. This decline in beneficial bacteria directly impacted a significant reduction in carbohydrate-related enzyme metabolism, crucial for the breakdown of intestinal carbohydrates into short-chain fatty acids that provide ample energy for the host [42]. The modifications in the gut microenvironment, mediated by T6SS, promoted the survival of *A. veronii* and certain *Helicobacter*, *Chlamydia*, and *Retrovirus* (Fig. S13). *Helicobacter* and *Retrovirus* infections elevate the host’s risk of developing cancer [58]. Therefore, it can be inferred that pathogens exploit T6SS to reshape the gut microenvironment, compete for host ecological niches and resources, and facilitate their own reproduction, leading to severe pathogenicity in the host.

Using unbiased secretomic analysis, we discovered novel effector proteins secreted by T6SS that disrupted the growth of *E. coli in vitro* and in physiological conditions. The mechanism of action involved disrupting rival bacteria’s cell membrane permeability, as shown by increased PI staining and membrane depolarization upon expression of specific effector proteins, like Tse1 and Tse3 (Fig. 7A–D). Furthermore, through targeted interaction with the cell wall, Tse2 has the ability to induce aberrant bacterial cell division (Fig. 7G and H). Homologous immunity proteins were co-evolved with effector proteins, providing protection to bacteria against their own toxicity. Our findings suggested that the Tse2 effector protein was the main driver of the competitive interaction between *A. veronii* and *E. coli*, while Tse1 and Tse3 had minimal effects. Deep sequencing analysis revealed a significant decrease in Kyoto Encyclopedia of Genes and Genomes (KEGG) metabolic pathways related to cell wall and cell membrane synthesis in *A. veronii* that possessed functional T6SS after infection. This correlation strongly supports our findings.

**Figure 8:**
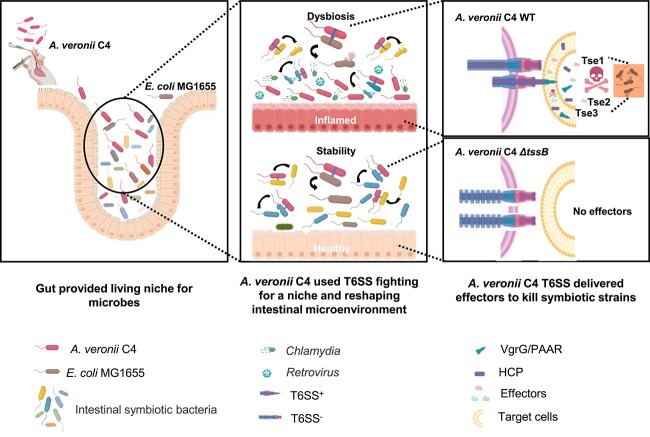
Mechanistic model of intestinal microenvironment remodeling; in healthy mice, the composition of the intestinal microbiota remained relatively stable; however, upon gavage infection with *A. veronii* (left), this pathogen recruited its T6SS to deliver various virulence factors into the commensal bacteria residing in the intestines, leading to their suppression or elimination (top middle and top right); consequently, a perturbation occurred in the original microbiota, resulting in dysbiosis within the intestinal microenvironment, thus facilitating the colonization of *A. veronii;* upon encountering the intestinal epithelial cells, *A. veronii* targeted them by releasing effector proteins through the T6SS, which induced intestinal inflammation; on the contrary, when the T6SS function was depleted (bottom middle and bottom right), *A. veronii* lost its ability to release effector proteins into the target cells; consequently, it was unable to eliminate the symbiotic bacteria or colonize itself within the ecological niche of the intestines; as long as the intestinal microenvironment kept stable and *A. veronii* failed to infect the intestines, the host’s intestinal health was preserved.

In summary, we employed T6SS-positive and T6SS-negative strains to infect the host and applied gut metagenomics to elucidate the reciprocal interaction between T6SS and the gut microbiota. Additionally, secretomic analysis was utilized to decipher the virulence effectors secreted by T6SS. The conclusive findings demonstrate that T6SS exerts a regulatory influence on the gut microbiota composition through the secretion of effector proteins Tse1, Tse2, and Tse3. This regulatory action leads to the suppression of beneficial bacteria growth and the remodeling of the gut microenvironment, favoring the survival of the T6SS-positive strain, and ultimately enhancing pathogenicity toward the host (Fig. 8). Aside from direct growth restriction and killing of intestinal bacteria, we found that the local immune status of the *A. veronii* infection site in the gut was also altered, highly correlating with T6SS. This stimulated immune response can also cause imbalance of gut microbiota. Thus, it is reasonable to propose that changes in gut microbiota result from both T6SS-mediated bacterial competition and the host immune response. Conversely, differences in the host’s response between the *A. veronii* C4 WT and the *ΔtssB* mutant (Fig. 2) might be a combined consequence of the microbiome remodeling and direct effects of the T6SS on host colonization. However, we cannot experimentally identify their contributions to T6SS-mediated immune stimulation. Our study provides a fresh perspective on the T6SS-mediated mechanisms underlying host pathogenesis, offering potential strategies for defense and treatment against T6SS-associated diseases by modulating the gut microbiota.

## Supplementary Material

Supplementary-2024_3_20_wrae053

## Data Availability

The experimental and computational data that support the findings of this research are available in this article and its supplementary information files, or upon request from the corresponding authors. The metagenomic data are available in NCBI BioProject (PRJNA1087356).
