# Supplementary material for: Role of Type VI secretion system in pathogenic remodeling of host gut microbiota during Aeromonas veronii infection
Source: ISME J. 2024 Mar 26;18(1):wrae053. doi: 10.1093/ismejo/wrae053 (PMC11014884; doi:10.1093/ismejo/wrae053)
Supplement: Supplementary-2024_3_20_wrae053 [file supplementary-2024_3_20_wrae053.docx]

**Role of type VI secretion system in pathogenic remodeling of host gut microbiota during *Aeromonas veronii* infection**

Xiaoli Jiang^1,4^, Hanzeng Li^1,4^, Jiayue Ma^1^, Hong Li^1^, Xiang Ma^1^, Yanqiong Tang^1^, Juanjuan Li^1^, Xue Chi^1^, Yong Deng^2^, Sheng Zeng^3^, Zhu Liu^1#^

**Supplementary Materials and Methods**

**Animal model**

Four-week-old male Kunming mice were purchased from the Hainan Pharmacology Research Center. The animals were housed at a controlled temperature of 20±2ºC and fed libitum with standard rat chow and distilled water. All mice involved in the experiment were euthanized by cervical dislocation.

**Plasmids and strain construction**

Knockout strain construction in *A. veronii*. Knockout strains in *A. veronii* were made using the *sacB* homologous recombination method[23].

HCP overexpression strain construction in *A. veronii*. Flag tags were incorporated at the C-terminus of the HCP coding region using PCR with genomic DNA from *A. veronii* WT strain as a template. The full-length flag-tagged HCP DNA fragment was then inserted into the linearized pBBR vector (digested with *Kpn* I and *Eco*R I) using the One Step CloneExpress kit (Vazyme, China). The resulting pBBR*-flag-hcp* plasmid was subsequently transformed into competent cells of *E. coli* WM3064 via heat shock. The HCP overexpression strains were created by conjugation with *E. coli* WM3064 carrying pBBR*-flag-hcp* plasmid, and were selected on solid medium containing both Ampicillin and Kanamycin and confirmed by colony PCR.

Fluorescent strain construction. To construct the pBBR*-eGFP*, the *eGFP* DNA fragment was amplified from the pUC*-eGFP* plasmid using PCR. The remaining steps are the same as above.

**Detection of HCP secretion efficiency by Western blots**

*A. veronii* derivatives with specified genotypes were cultivated in liquid culture medium at 30°C until reaching an optical density OD_600nm_=1. The supernatant was filtered through a 0.22 µm filter to remove any residual bacteria. To enrich the secreted proteins, the filtrate was subjected by TCA precipitation[24]. The resolubilized secreted proteins and the resulting pellets were separately suspended in 50 µl of 1× SDS loading buffer and boiled for 30 min before being loaded onto a 12% SDS-PAGE gel for electrophoresis. Standard western blotting techniques were executed to evaluate the presence of the T6SS substrate HCP in the supernatant and pellet samples. An anti-flag antibody (Sangon, China) was used as the primary antibody, followed by incubation with a FITC-conjugated anti-mouse IgG antibody (Sangon, China). The blots were subsequently scanned and analyzed using a multifunctional biomolecular Typhoon imager FLA9500 (Cytiva, USA).

Preparation of reference antibody *A. veronii* GAPDH. The antigenic epitope peptide of GAPDH protein was synthesized by the company (Sangon, China). The mice were weighed and immunized with a dosage of 0.5 mg/kg. The synthesized antigen peptide was mixed with an equal amount of Freund's complete adjuvant, vigorously shaken until emulsified without phase separation. The injection site was shaved, disinfected with 75% alcohol, and the emulsion was extracted using a syringe for subcutaneous injection in the mice. After 10 days, a second immunization was performed with the same dosage, but the antigen peptide was emulsified with Freund's incomplete adjuvant. Subsequently, injections were administered every 7 days, for a total of 5 times immunizations. Finally, the prepared polyclonal antiserum was evaluated for its potency. It was stored at -80°C for future use.

**Cytotoxicity assay**

To assess the cytotoxicity of *A. veronii* derivatives, we cultured the immortalized mouse macrophage cell line RAW 264.7 in DMEM supplemented with standard supplements, including 10% FBS, L-glutamine, antibiotics (Penicillin/Streptomycin). For each well in 96-well plates, we seeded 5 × 10^4^ RAW 264.7. Subsequently, the cells were incubated with *A. veronii* (5 × 10^5^ CFU/ml, the multiplicity of infection was 10) for 3 h. Following the incubation period, cell viability was measured using the CCK-8 cell viability kit (Servicebio, China). Uninfected RAW 264.7 cells served as negative controls for comparison.

***In vivo* colonization of *A. veronii* derivatives and pathological assessment**

Mice were separately infected with 10^9^ CFU/g of wild-type *A. veronii* (WT), *ΔtssB*, *ΔtssB::tssB* strain, and PBS control. After a 2-day infection period, the kidney, cecum, and colon were dissected, weighed, and divided into two parts. One part of the tissues was fixed with 4% paraformaldehyde (PFA) for pathological analysis, while the other part was homogenized using a tissue grinder. The resulting supernatants were plated onto LB agar plates containing Amp (50 µg/ml) and Kan (50 µg/ml), and incubated at 37°C for 12 h to quantify CFU of each bacterial strain.

For pathologic analysis, PFA-fixed kidney and colon samples (0.5 cm in length) were treated with 10% formalin for 24 h, followed by transfer to 70% ethanol. The samples were embedded and sliced using paraffin sectioning. The sliced tissues were then subjected to hematoxylin and eosin (H&E) staining and imaged using a Microscope (Servicebio, China).

**Competition assay between bacteria**

To evaluate whether *A. veronii* participated in bacterial interspecies competition, *E. coli* BL21, *E. coli* DH5α and *E. coli* MG1655 were selected as prey *A. veronii* derivatives (WT, *ΔtssB*, and *ΔtssB::tssB*) were prepared in overnight liquid cultures containing ampicillin (100 µg/ml) and kanamycin (50 µg/ml) and served as attackers. Both prey and attacker cells were adjusted to an OD of 1 at 600_nm_. Attacker and prey strains were mixed in a 1:1 ratio and spotted onto solid LB medium without antibiotics, followed by co-incubation for 6 h. Prey viability was assessed using a colony formation assay and flow cytometry.

For anaerobic *in vitro* competition experiments, all operations are performed at the anaerobic operating station (Coy Vinyl Anaerobic Chambers, America). The competitive experimental methods are the same as above. Both *Lachnospiraceae bacterium* and *A. veronii* use GAM medium to grow, inoculate and resuscitate in anaerobic incubator. The Attacker and prey strains were mixed 1:1 in the 96-well plate for 24 h. The number of colony recovery was then measured.

For *in vivo* assessment, the mammalian commensal *E. coli* MG1655 was used as prey. Host mice were pretreated with a combination of antibiotics [Ampicillin (1 mg/ml), Vancomycin (0.5 mg/ml), Neomycin (1 mg/ml), Metronidazole (1 mg/ml), and Gentamicin (1 mg/ml)] for two days to purge the gut microbiota. *E. coli* MG1655 at a concentration of 10^9^ CFU was colonized in the mice intestines via gastric gavage for 24 h. Subsequently, *A. veronii* derivatives (WT or *ΔtssB*) at a concentration of 10^9^ CFU were introduced. After 24 h of infection, the mice were euthanized, and intestinal feces were collected to measure MG1655 viability as CFU per gram of feces, cecum and colon.

**Expression vector construction and growth inhibition experiment of effector proteins**

The coding regions of presumed effector proteins Tse1, Tse2, and Tse3 were amplified by PCR, using specific primers and the *A. veronii* genome as a template. Following amplification, the DNA fragments were fused with signal peptides and subsequently inserted into plasmid pET25b. The resulting plasmid was then transformed into *E. coli* BL21 to enable directed expression in both the periplasmic and cytoplasmic compartments. To initiate the growth inhibition experiment, single transformant was carefully selected and cultured overnight at 37°C in LB medium supplemented with Ampicillin. Subsequently, the bacterial suspension was adjusted to a concentration of 10^8^ CFU/ml. A volume of 2.5 μl of the suspension was then carefully spotted onto solid medium containing both Ampicillin and IPTG. The plates were incubated at 37°C for 24 h.

Construction of a plasmid co-expression system was performed as follows. Target fragments of immune proteins Tsi1, Tsi2, and Tsi3 were amplified from *A. veronii* genomic DNA using specific primers. The expression vector chosen for this purpose was the pBBR plasmid, which contains constitutive expression promoters. One Step CloneExpress ligation kit (Vazyme, China) was employed to seamlessly connect the vector with the amplified fragments, leading to the generation of recombinant vectors, namely pBBR*-tsi1*, pBBR*-tsi2*, and pBBR*-tsi3*. Subsequently, these vectors, along with the respective effector protein expression vectors mentioned earlier, were co-transformed into competent BL21 cells to allow for protein expression. The co-transformed cells were inoculated with an initial OD_600nm_=0.01 and incubated at 37°C for 2 h. Afterward, 0.05 mM IPTG was added for induction, and the growth of each group was monitored using a spectrophotometer (BioTek Synergy H1, USA).

**Membrane** **potential measurement**

To determine the impact of effector proteins on reducing the membrane potential, a mitochondrial membrane potential detection kit (JC-1) was applied (Beyotime, China). The strains expressing effector proteins in the periplasmic space were cultured overnight, and then diluted to a concentration of 10^7^ CFU/ml. These diluted cultures were transferred to a 96-well plate and subjected to growth on a shaker at 37°C for 2 h. Subsequently, IPTG was added to a final concentration of 1 mM, and the cells were grown for another 2 h. The bacterial pellets were washed twice with PBS buffer, and then resuspended in 500 μl of JC-1 working solution. The suspension was then incubated at 37°C in darkness for 20 min. As a positive control, carbonyl cyanide 3-chlorophenylhydrazone (CCCP) was added to *E. coli* expressing the empty plasmid pET25b at a final concentration of 10 μM, five minutes prior to JC-1 staining. The samples were analyzed using a Beckman CytoFlex flow cytometer. At least 50,000 bacteria were gated based on forward and side scatter parameters. The JC-1 dye was excited using a blue laser (488 nm), and the emissions were detected using 530/30 (green) and 590/40 (red) filters. The red/green ratio of the gated bacteria was calculated by determining the geometric mean fluorescence intensity (MFI) for each channel using FlowJo software.

**Measurement of membrane permeability**

To determine whether the effector proteins caused an increase in membrane permeability, cultures of *E. coli* BL21 were grown and treated in a similar manner as described for membrane potential measurement. A 200 μl sample was transferred to a 96-well plate and treated with 10 μg/ml propidium iodide (PI)[25].

**Purification of Tse2 protein**

The recombinant Tse2 was purified by nickel affinity method. BL21 (plysS) -pet28a-Tse2 was inoculated, and 0.4 mM IPTG was added when the strain grew to OD600nm=0.4 and induced at 16℃ for 18 h；After the bacteria were collected, the bacteria were re-suspended with a 10 mL balance buffer (pH=7.4, PBS buffer, 0.5 M Nacl, 20 mM imidazole), and the cells were broken by ultrasound at low temperature. The supernatant was collected by centrifugation at 12000 rpm at 4℃ for 10 min；The nickel column was balanced by 10 mM buffer at low temperature for 1 h; The protein was eluted with 100 mM imidazole. The eluted protein sample was stored in the refrigerator at 4℃ or immediately added with 1×SDS loading buffer, and then run SDS-PAGE gel electrophoresis to verify whether the protein was purified successfully.

**Lysis assay**

After inoculation with *E.coli* BL21 and overnight culture, the concentration of bacteria was 10^8^ CFU/mL. Experimental group and control group were set up, 96-well plates were used, 100 µL bacterial solution was taken into each hole, and the absorbance value was measured at A_600nm_ for 0 min. PBS was negative control, 1 mg/mL Lysozyme was positive control, and 1 mg/mL Tse2 was experimental group, and 10 µL of Polymyxin B (4 mg/mL) was added to all of them. Put it in an incubator at 37℃ for 10 min and test the absorbance value of A_600nm_ for 10 min. The bacterial cracking rate was calculated by the formula: $\frac{[(A600 at 10min- A600 at 0min)]}{A600 at 0min}$×100%.

**Fluorescence microscopy of cell membrane**

*E. coli* BL21*::Empty* and BL21*::Tse2^peri^* were inoculated using the aforementioned method, with an initial OD600_nm_ of 0.01. After incubating at 37°C for 2 h, 1 mM IPTG was added to induce expression for an additional 2 h. The final concentration of FM4-64 was 10 µM by adding 1 µL of FM4-64 dye, and the dye was dyed for 10 min. 10 µL of each stained bacterial solution was placed on a slide under a high-resolution confocal laser microscope (Nikon, Japan), and the cell morphology was observed by cy5.5 fluorescence channel.

**Transmission electron microscopy (TEM) imaging**

*E. coli* BL21*::Empty* and BL21*::Tse2^peri^* were inoculated using the aforementioned method, with an initial OD600_nm_ of 0.01. After incubating at 37°C for 2 h, 1 mM IPTG was added to induce expression for an additional 2 h. Subsequently, 200 μl of bacterial suspension was taken, and after centrifugation at 4000 rpm for 3 min, the supernatant was discarded. The pellet was then fixed with 2.5% glutaraldehyde electron microscopy fixative for 1 h, followed by three washes with PBS. Next, 10 μl of bacterial suspension was dropped onto a copper grid, allowed to air dry naturally, and the bacterial morphology was captured using TEM ([Hitachi](javascript:;), Japan).

**Supplementary Figures**

**
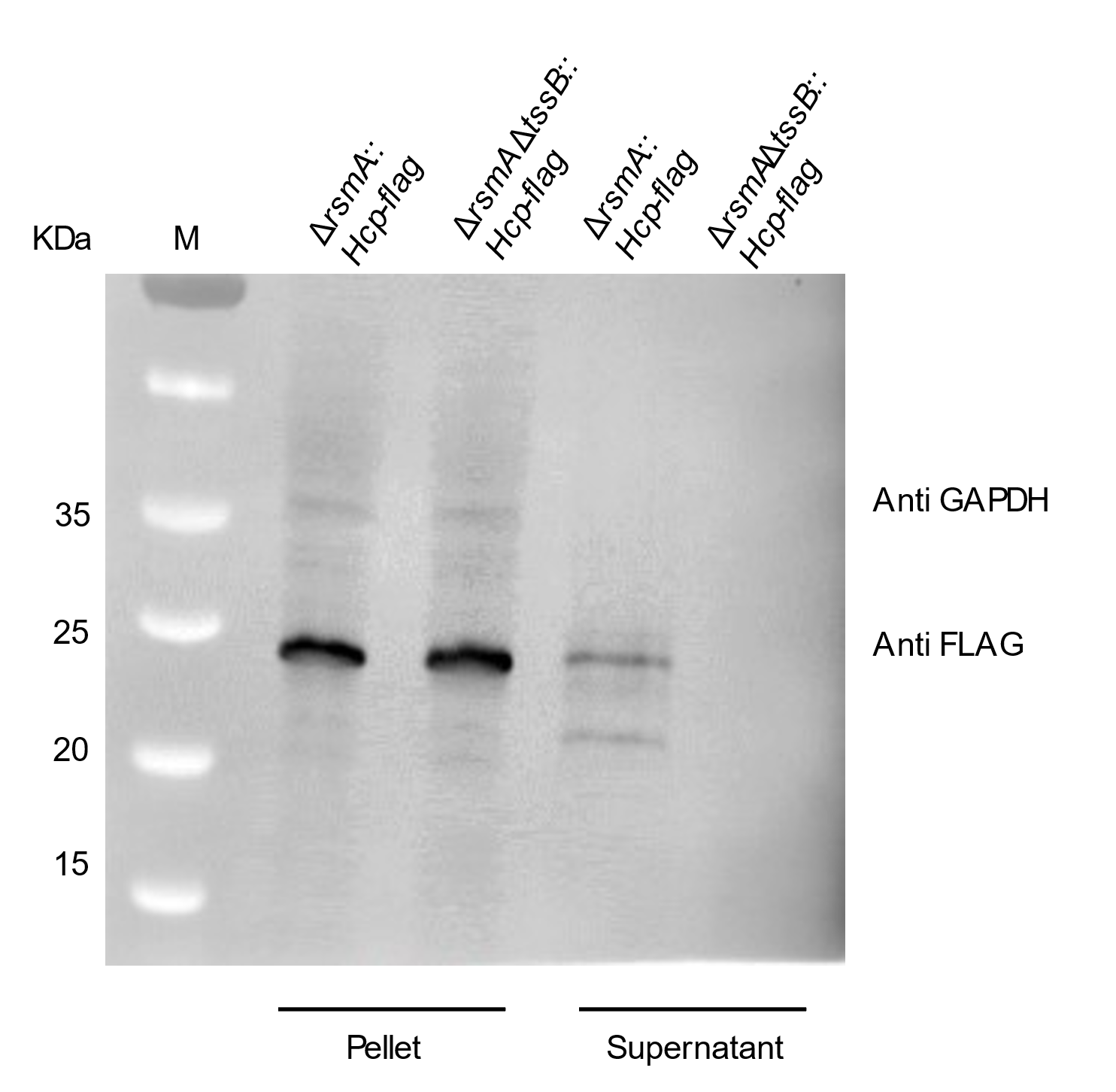
**

**Figure S1.** Western blot analysis of HCP secretion in the indicated strains, including flag-tagged HCP expressed in *ΔrsmA* or *ΔrsmAΔtssB*. HCP was detected using an anti-flag antibody in pelleted cells or supernatants. The housekeeping non-secreted protein GAPDH served as a loading control.

**
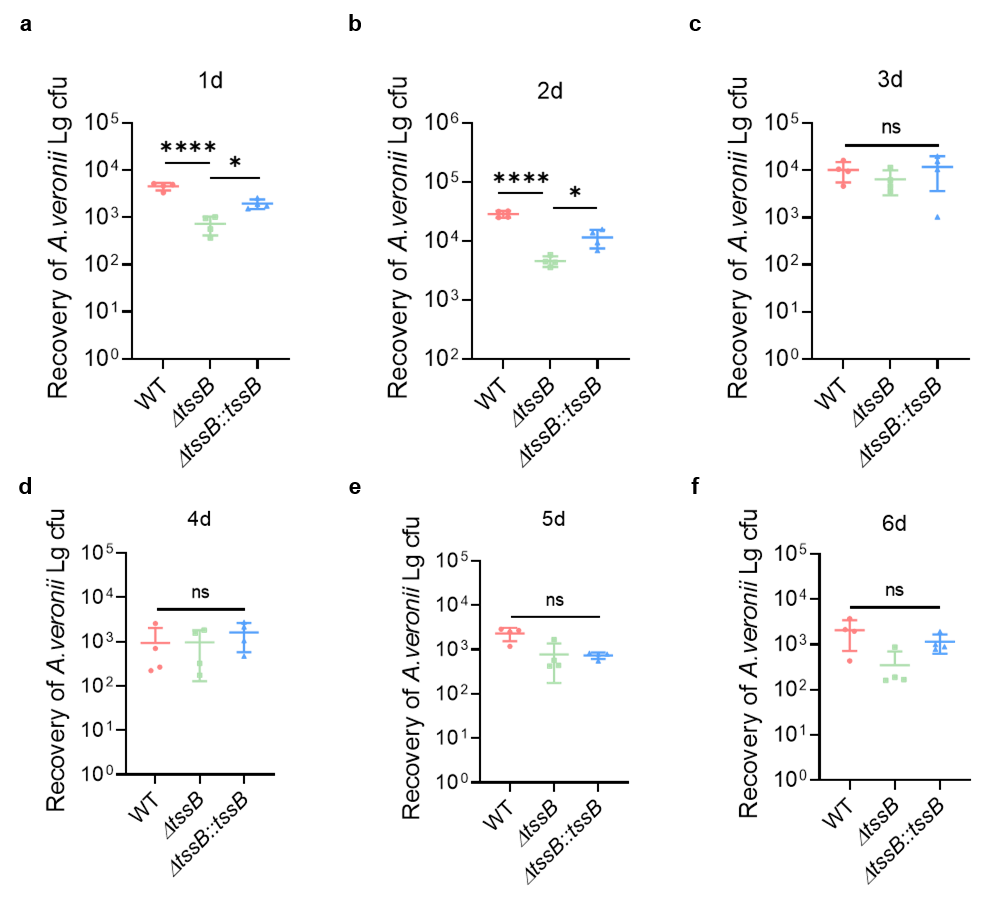
**

**Figure S2.** Temporal variations in the fecal colony counts of *A. veronii* C4. **a-f** The number of *A. veronii* C4 colonies in mouse feces were monitored over a period of 6 consecutive days. Statistical significance was determined by one-way ANOVA test: *p*<0.0001(****), 0.01<*p*<0.05(*) indicated significance, and "ns" signified no significant difference. n=5 for each group.

**
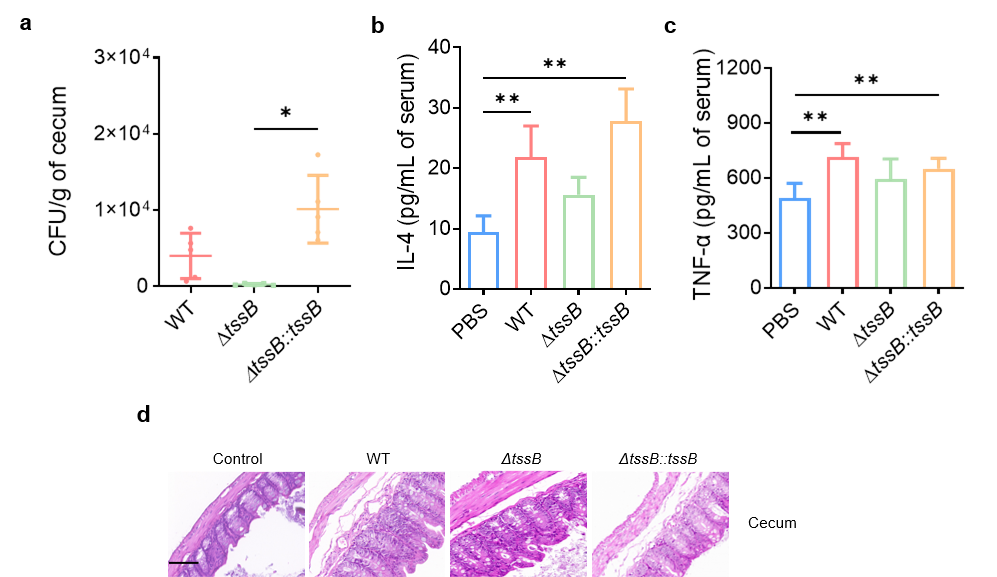
**

**Figure S3.** **a** Quantification of colony forming units (CFU) of WT, *ΔtssB*, and *ΔtssB::tssB* strain isolated from three tissues of infected mice including cecum. **b, c** Levels of cytokines in the serum. Assessment of serum cytokine levels in mice infected with WT, *ΔtssB*, *ΔtssB::tssB*, or saline control (PBS). The cytokine levels were quantified and presented as mean±SD, with error bars representing the standard deviation. One-way ANOVA testing was applied to determine the statistical significance, with *p*<0.01(**), 0.01<*p*<0.05(*) indicated significance, and “ns” denoted no significant difference. **d** Histological examination (HE) of pathological alteration in the cecum of mice infected with WT, *ΔtssB*, and *ΔtssB::tssB* strains of *A. veronii*. The sample size for each group was n=5. Scale bars were shown with indicated length (50 µm).

**
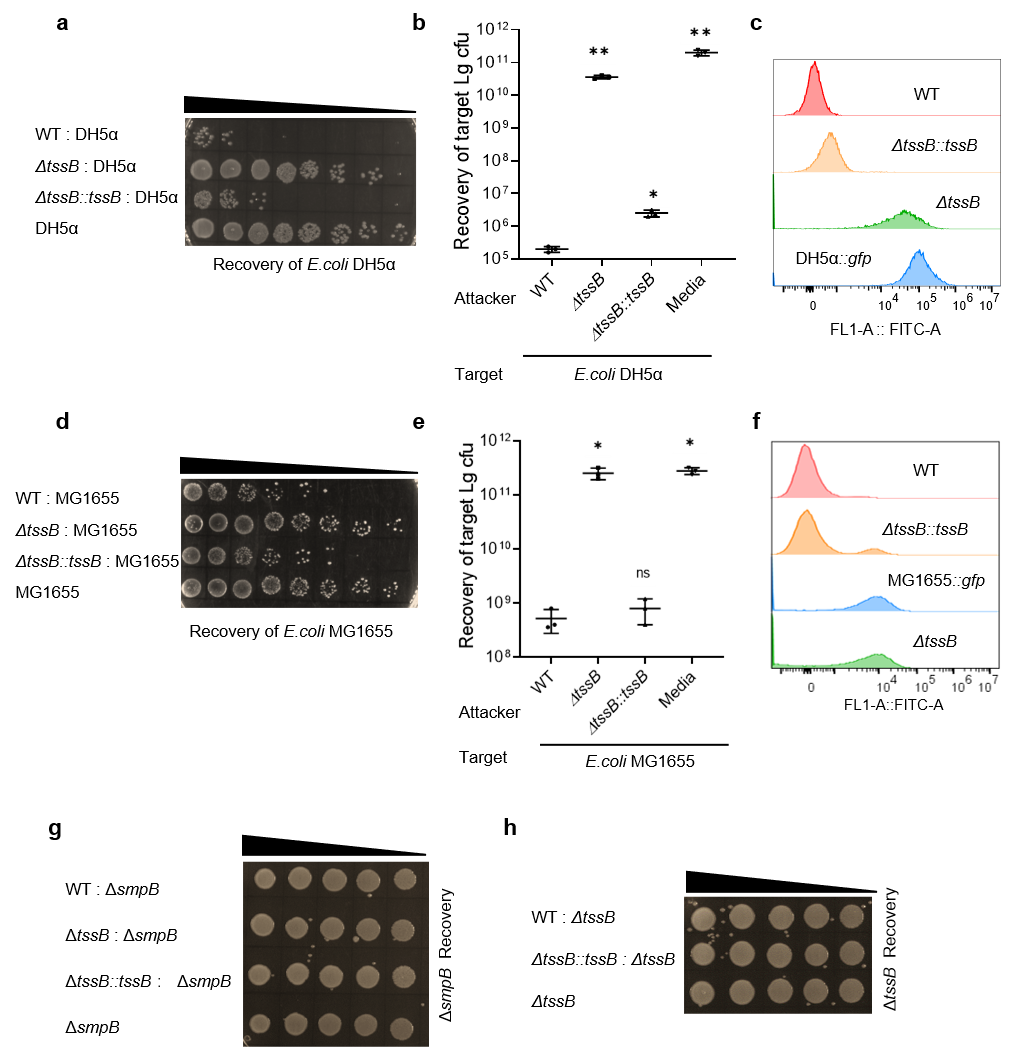
**

**Figure S4. a-f** The growth inhibition of *A. veronii* on *E. coli* DH5α and *E. coli* MG1655. **a, d** Representative images of *E. coli* DH5α and *E. coli* MG1655 recovery assay after co-incubation with serial dilutions of *A. veronii* variants, including WT, *ΔtssB*, *ΔtssB::tssB*, and *E. coli* DH5α, *E. coli* MG1655 only. **b, e** Quantification of surviving rates of *E. coli* DH5α and *E. coli* MG1655 after co-incubation with *A. veronii* (n=3). **c, f** Flow cytometry analysis was performed to evaluate the survival of *E. coli* DH5α and *E. coli* MG1655, which was indicated by eGFP expression, after co-incubation with different variants of *A. veronii*, by using the FITC channel(n=3). **g, h** Intra-species competition among *A. veronii*. *In vitro* competition assays showing that the absence of outcompeting WT by *ΔsmpB* or *ΔtssB* mutants. One-way ANOVA testing was applied to determine the statistical significance, *p*<0.01(**), 0.01<*p*<0.05(*) indicated significance and “ns” denoted no significant difference.


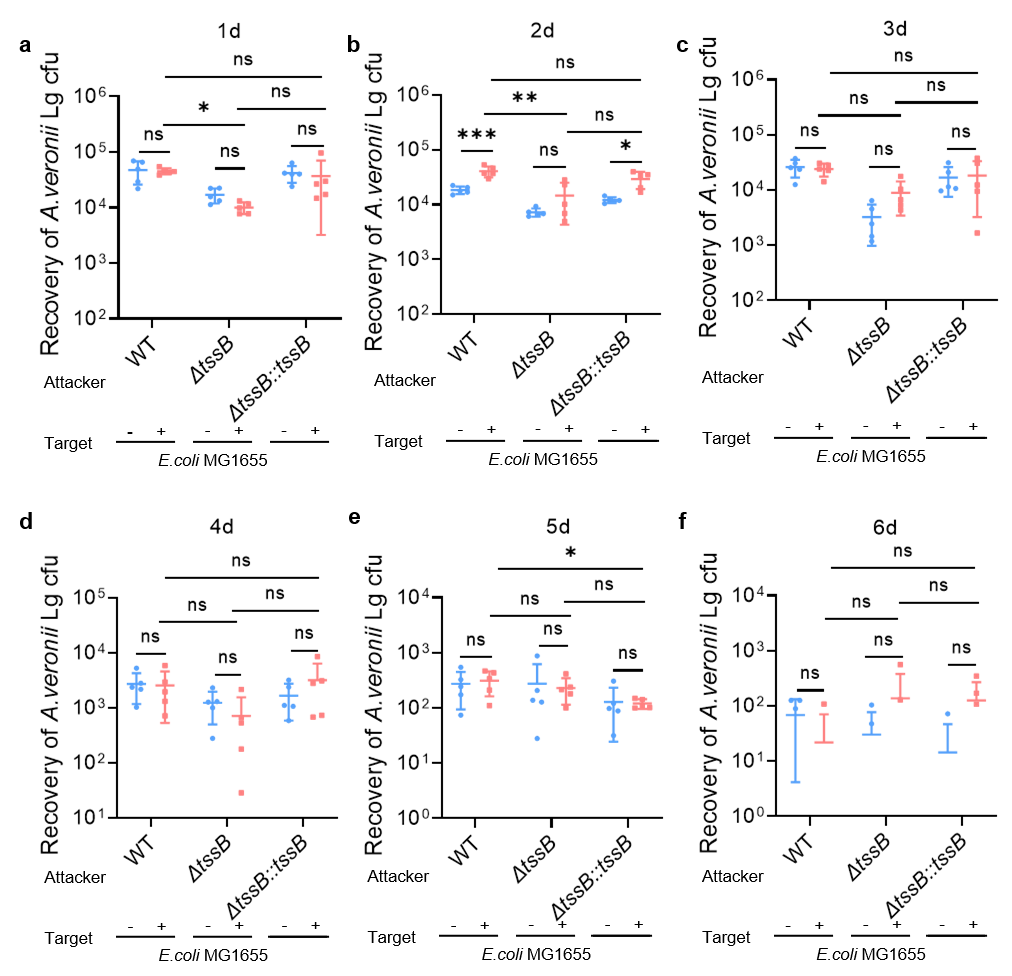


**Figure S5.** The number of *A. veronii* C4 in feces was measured after competition. **a-f** After *in vivo* competition, the survival number of *A.veronii* C4 in feces was measured every day for 6 consecutive days. Statistical significance was determined by one-way ANOVA test: *p*<0.0002(***), 0.01<*p*<0.05(*) indicated significance, and "ns" signified no significant difference. n=5 for each group.


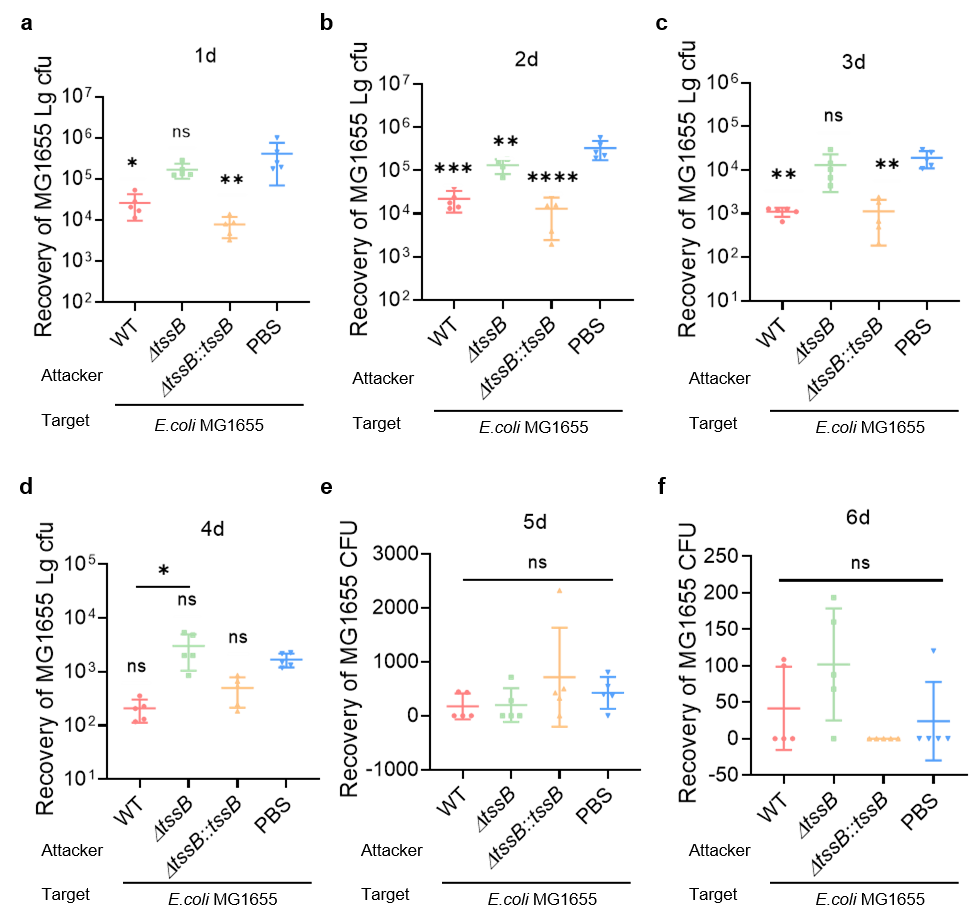


**Figure S6.** The number of *E.coli* MG1655 in feces was measured after competition. **a-f** After *in vivo* competition, the survival number of *E.coli* MG1655 in feces was measured every day for 6 consecutive days. Statistical significance was determined by one-way ANOVA test: *p*<0.0001(****), *p*<0.0002(***), *p*<0.01(**) and 0.01<*p*<0.05(*) indicated significance, and "ns" signified no significant difference. n=5 for each group.


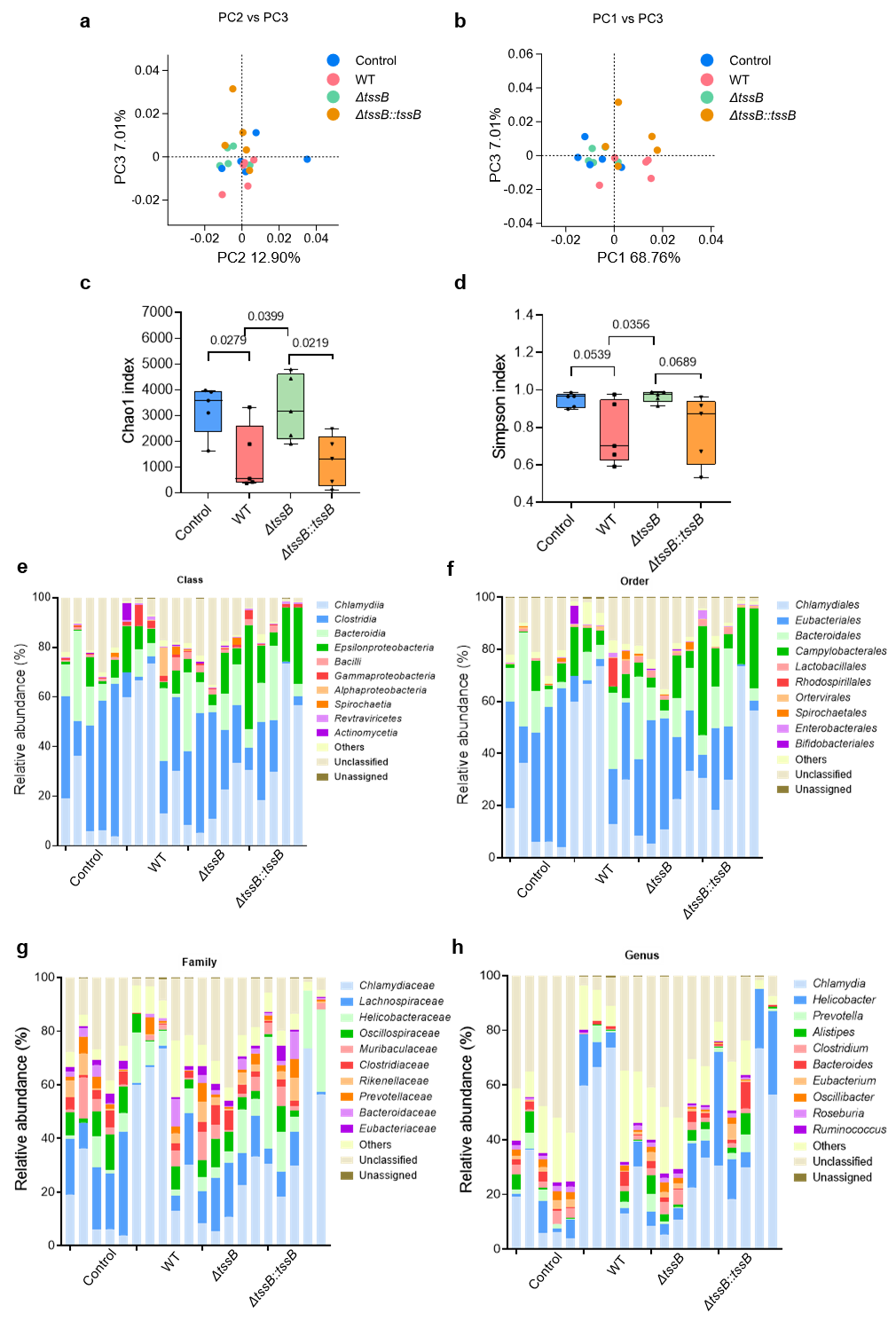


**Figure S7.** T6SS reshapes host gut microbiota during *A. veronii* infection. **a, b** Data analysis using PCA. Visual representation was achieved using colored ellipses and points. **c, d** Demonstrating alpha diversity using Chao1 and Simpson indices. The statistical significance was determined by *t*-test. The significance levels were denoted as follows: *p*<0.01(**), 0.01<*p*<0.05(*) indicated significance, and "ns" signified no significant difference. **e-h** Distribution of composition at the Class**(e)**, Order**(f)**, Family**(g)**, and Genus**(h)** levels.

**
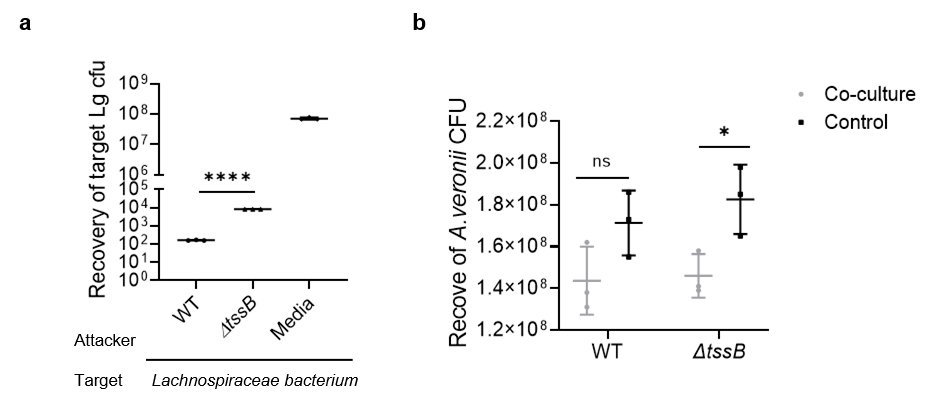
 Figure S8.** Competition between *A. veronii* C4 and *Lachnospiraceae bacterium* *in vitro*. **a** Quantification of surviving rates of *Lachnospiraceae bacterium* after co-incubation with *A. veronii* C4 (n=3). **b** Quantification of surviving rates of *A. veronii* C4 after co-incubation with *Lachnospiraceae bacterium* (n=3). Statistical significance was determined by one-way ANOVA test: *p*<0.0001(****), 0.01<*p*<0.05(*) indicated significance, and "ns" signified no significant difference.

**
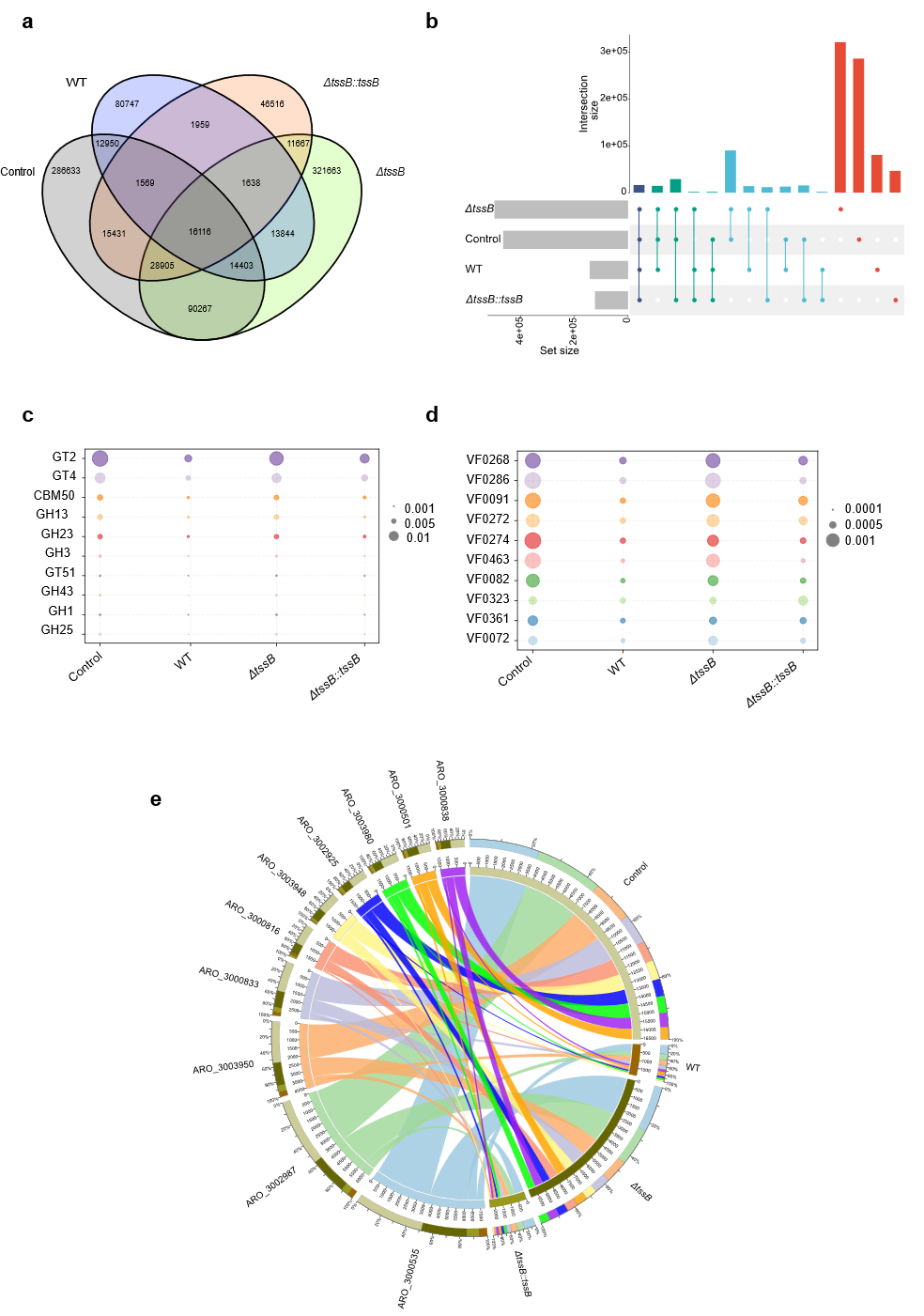
**

**Figure S9.** Inter-group functional difference analysis**.** **a, b** Differences between Gene Number Venn Diagram and Upset Plot. **c, d** Functional differences, CAZy **(c)** and VFDB **(d)** analysis. **e** CARD antibiotic resistance gene composition circular plot. The outer circle's right half represented the samples or groups, while the left half represented the annotated types of resistance genes. The scale indicated the proportion of abundance. The inner circle's ribbons connected the resistance genes with the samples or groups, revealing the composition of resistance gene functions within the samples/groups and the composition of samples/groups with resistance genes. The width of the ribbons represented the distribution proportion.


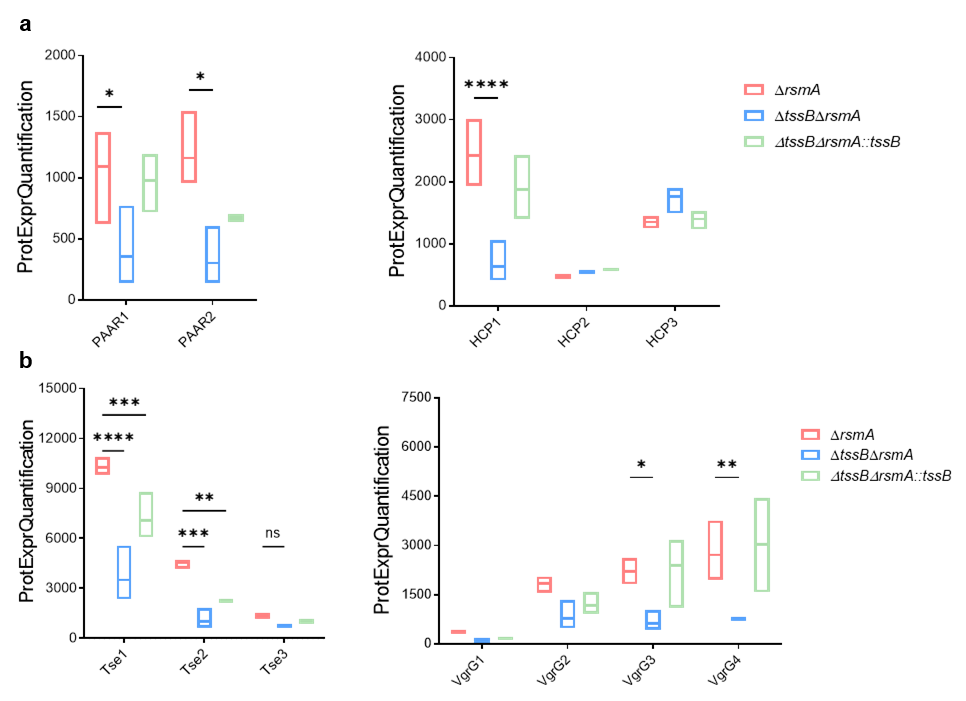


**Figure S10. a, b** Quantification of protein levels in the secretome of the indicated strains by mass spectrometry. Data were shown as mean±SD, with error bars standed for the standard deviation. Statistical significance was determined by one-way ANOVA test: *p*<0.0001(****), *p*<0.0002(***), *p*<0.01(**) and 0.01<*p*<0.05(*) indicated significance. n=3 for each group.


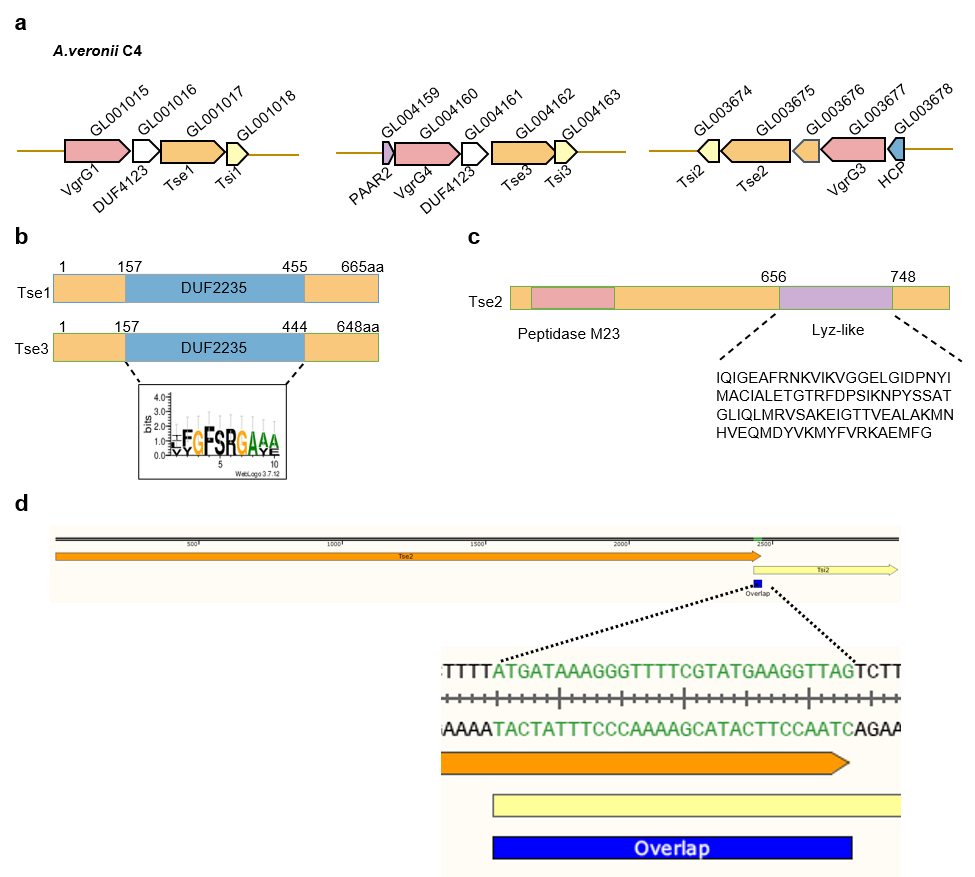


**Figure S11. a** Gene cluster encoding putatively secreted effector proteins by T6SS in *A. veronii*. **b, c** Schematic illustration of protein domain structures for two conserved effector proteins, Tse1 and Tse3, highlighting the consensus sequence within the DUF2235 domain **b**, **c** The probability of amino acids at each position were represented as heights of corresponding amino acid code. Composition of Tse2 protein domains. **d** Nucleotide sequence composition of *tse2-tsi2* genes.

**
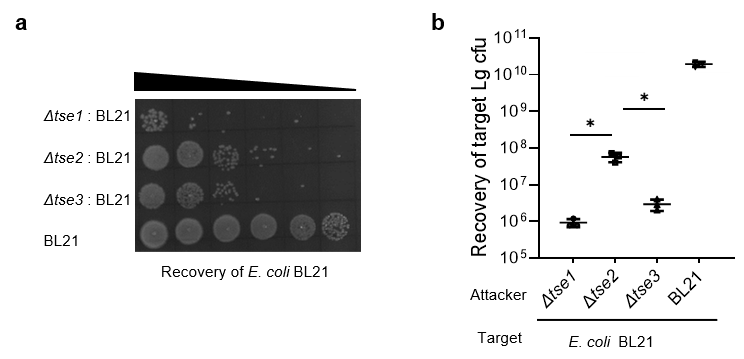
**

**Figure S12. a, b** Inter-bacterial competition and functional validation of identified effector proteins in *A. veronii*. Representative images of BL21 recovery assay after co-incubation with serial dilutions of *A. veronii* variants, including *Δtse1*, *Δtse2*, *Δtse3*, and BL21 only a. **b** Quantification of surviving rates of *E. coli* BL21 after co-incubation with aforementioned strains. Statistical significance was determined by one-way ANOVA test: *p*<0.01(**) and 0.01<*p*<0.05(*) indicated significance. n=3 for each group.


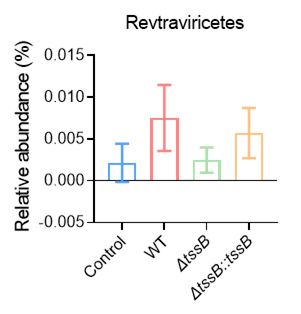


**Figure S13.** Comparison of inter-group differences in viral distribution. Performing inter-group differential analysis of viral abundance in the sequencing results.

**Supplementary Tables**

**Table S1. Bacterial strains and plasmids used in this study.**

| **Strain or Plasmid** | **Description** | **Sources** |
| --- | --- | --- |
| ***A.veronii* C4** | **Cultured in 30℃.** |  |
| WT | Wild type; Amp resistance. | Lab stock |
| *ΔtssB* | Amp resistance, deleted *tssB* gene from WT. | This study |
| *ΔtssB::tssB* | Amp and Kan resistance, deleted *tssB* gene from WT and complemented with functional *tssB* gene. | This study |
| *ΔrsmA* | Amp resistance, deleted *rsmA* gene from WT. | This study |
| *ΔrsmAΔtssB* | Amp resistance, deleted *rsmA* and *tssB* gene from WT. | This study |
| *ΔrsmAΔtssB::tssB* | Amp and Kan resistance, deleted *rsmA* and *tssB* gene from WT and complemented with functional *tssB* gene. | This study |
| *ΔsmpB* | Amp resistance, deleted *smpB* gene from WT. | Lab stock |
| *ΔsmpB::smpB* | Amp and Kan resistance, deleted *smpB* gene from WT and complemented with functional *smpB* gene. | Lab stock |
| *Δtse1* | Amp resistance, deleted *tse1* gene from WT. | This study |
| *Δtse1Δtsi1* | Amp resistance, deleted *tse1* and *tsi1* gene from WT. | This study |
| *Δtse1Δtsi1::tsi1* | Amp and kan resistance, deleted *tse1* and *tsi1* gene from WT and complemented with functional *tsi1* gene. | This study |
| *Δtse2* | Amp resistance, deleted *tse2* gene from WT. | This study |
| *Δtse2Δtsi2* | Amp resistance, deleted *tse2* and *tsi2* gene from WT. | This study |
| *Δtse2Δtsi2::Δtsi2* | Amp and Kan resistance, deleted *tse2* and *tsi2* gene from WT and complemented with functional *tsi2* gene. | This study |
| *Δtse3* | Amp resistance, deleted *tse3* gene from WT. | This study |
| *Δtse3Δtsi3* | Amp resistance, deleted *tse3* and *tsi3* gene from WT. | This study |
| *Δtse3Δtsi3::Δtsi3* | Amp and Kan resistance, deleted *tse3* and *tsi3* gene from WT and complemented with functional *tsi3* gene. | This study |
| ***E.Coli*** | **Cultured in 37℃.** |  |
| *E.Coli* WM3064 | Dap nutritional deficiency. | Lab stock |
| *E.Coli* DH5α | Used for plasmid cloning and for protein expression. | Lab stock |
| *E.Coli* MG1655 | Intestinal symbiotic bacteria, used for *in vivo* bacteria competition, Rif resistance. | BNCC |
| *E.Coli* BL21（DE3) | Used for plasmid cloning and for protein expression. | Lab stock |
| *E.Coli* BL21（DE3)pLysS | Used for virulence protein expression. | Lab stock |
| **Intestinal probiotic** | **Strictly anaerobic culture at 37℃.** |  |
| *Lachnospiraceae bacterium* | Intestinal probiotic, Kan resistance. | BNCC |
| *Blautia coccoides* | Intestinal probiotic. | BNCC |
| *Clostridium beijerinckii* | Intestinal probiotic. | BNCC |
| **Plasmid** |  |  |
| pBBR-MCS-2 | Shuttle plasmid, used for protein expression, Kan resistance. | Lab stock |
| pBBR-*tssB* | Contains ORF region of *tssB*. | This study |
| pBBR- *hcp-flag* | Contains ORF region of *hcp-flag*. | This study |
| pBBR-*eGFP* | Contains ORF region of *eGFP*. | This study |
| pBBR-*tsi1* | Contains ORF region of *tsi1*. | This study |
| pBBR-*tsi2* | Contains ORF region of *tsi2*. | This study |
| pBBR-*tsi3* | Contains ORF region of *tsi3*. | This study |
| pET25b-SP | Contains PelB signaling peptide, Amp resistance, used for periplasmic protein expression. | Hunan Fenghui Biotechnology |
| pET25b  (pEmpty) | Do not contain PelB signaling peptide, Amp resistance, used for cytoplasmic protein expression. | This study |
| pET25b-*tse1*  (pTse1^Cyto^) | Contains ORF region of *tse1*, enables the expression of *Tse1* protein in the cytoplasm. | This study |
| pET25b SP-*tse1*  (pTse1^Peri^) | Contains ORF region of *tse1*, enables the expression of Tse1 protein in the periplasmic. | This study |
| pET25b-*tse2*  (pTse2^Cyto^) | Contains ORF region of *tse2*, enables the expression of Tse2 protein in the cytoplasm. | This study |
| pET25b SP-*tse2*  (pTse2^Peri^) | Contains ORF region of *tse2*, enables the expression of Tse2 protein in the periplasmic. | This study |
| pET25b-*tse3*  (pTse3^Cyto^) | Contains ORF region of *tse3*, enables the expression of Tse3 protein in the cytoplasm. | This study |
| pET25b SP-*tse3*  (pTse3^Peri^) | Contains ORF region of *tse3*, enables the expression of Tse3 protein in the periplasmic. | This study |
| pET28a | Used for protein expression, contains His tag, Kan resistance. | Lab stock |
| pET28a-*tse2* | Contains ORF region of *tse2*. | This study |
| pET28a-*tse2* | Contains ORF region of *tse2*. | This study |
| pRE112 | Suicide plasmid for homologous recombination exchange to construct knockout strains, Chl resistant. | Lab stock |
| pRE112-*ΔtssB* | Used to knock out the *tssB* gene in *A.veronii* C4. | This study |
| pRE112-*ΔrsmA* | Used to knock out the *rsmA* gene in *A.veronii* C4. | This study |
| pRE112-*Δtse1* | Used to knock out the *tse1* gene in *A.veronii* C4. | This study |
| pRE112-*Δtse1Δtsi1* | Used to knock out the *tse1* and *tsi1* gene in *A.veronii* C4. | This study |
| pRE112-*Δtse2* | Used to knock out the *tse2* gene in *A.veronii* C4. | This study |
| pRE112-*Δtse2Δtsi2* | Used to knock out the *tse2* and *tsi2* gene in *A.veronii* C4. | This study |
| pRE112-*Δtse3* | Used to knock out the *tse3* gene in *A.veronii* C4. | This study |
| pRE112-*Δtse3vtsi3* | Used to knock out the *tse3* and *tsi3* gene in *A.veronii* C4. | This study |

**Table S2. The primers utilized in this study.**

| **Primer name** | **Sequence (5’-3’)** | **Purpose** |
| --- | --- | --- |
| F1-*tssB* | catgaattcccgggagagctcTTATTGTGAAGTCATCGTGCGAG | Construction of ΔtssB |
| R1-*tssB* | accatTGTGGCGCCGTCAATTCC |  |
| F2-*tssB* | gtacaagtaaTCGGCGGGTTGTATTGACTG |  |
| R2-*tssB* | caagcttcttctagaggtaccCCTTGGTAGCGTGCAGCACC |  |
| F0-*tssB* | GGCTTAACGACTGATTTTGACG |  |
| R0-*tssB* | GTTGAATAGACATGCTTGTAGAGACC |  |
| F-*ptss*B | CCGGAATTCCGGGATGGCTCAACTGTAATATC | Construction of pBBR*-tssB* and *ΔtssB::tssB* |
| R-*ptssB* | TCACTCGGCAGGCTTGATCAA |  |
| F1-*rsmA* | catgaattcccgggagagctcGACCAGATCGCCGCAGAGC | Construction of *ΔrsmA* |
| R1-*rsmA* | ttcttgcacgaccggtatTCCTGTGGCACTCCCGTACA |  |
| F2-*rsmA* | gaATACCGGTCGTGCAAGAAGTTC |  |
| R2-*rsmA* | caagcttcttctagaggtaccCTCAACCCCCACGCAGGG |  |
| F0-*rsmA* | CAGCAGGTGCAGGTCGTGGTTG |  |
| R0-*rsmA* | AGATGTGGTGATGATGCTGG |  |
| F-*phcp* | ATGGATTACAAGGACGACGATGACAAGCCAACTCCATGTTATATCAGCATC | Construction of pBBR*-hcp-Flag* |
| R-*phcp-flag* | caagcttcttctagaggtaccCAGAAAAGCTCCAGCCAACCC |  |
| F-*eGFP* | GGGGTACCCATGGTGAGCAAGGGCGAGGAG | Construction of pBBR-e*GFP* |
| R-*eGFP* | CGGAATTCTTACTTGTACAGCTCGTCCATG |  |
| F-*tse1* | CTTTAAGAAGGAGATATACATATGGCTATCAGTGCCCACTG | Construction of pET-*SP-tse1* and pET -*tse1* |
| F-SP-*tse1* | CTGCCCAGCCGGCGATGGCCATGGCTATCAGTGCCCACTG |  |
| R-*tse1* | GAATTAATTCCGATATCCATTACCTCCTGATTACCGATC |  |
| F-*tse2* | CTTTAAGAAGGAGATATACATATGGCTATCAGTGCCCACTGC | Construction of pET-*SP-tse2* and pET -*tse2* |
| F-SP-*tse2* | CTGCCCAGCCGGCGATGGCCATGGCTATCAGTGCCCACTGC |  |
| R-*tse2* | GAATTAATTCCGATATCCATTTTACCCCCCTCCAAGTTAC |  |
| F-*tse3* | CTTTAAGAAGGAGATATACATGTGATCCTTGAACATCGAAAC | Construction of pET-*SP-tse3* and pET -*tse3* |
| F-SP-*tse3* | CTGCCCAGCCGGCGATGGCCGTGATCCTTGAACATCGAAAC |  |
| R-*tse3* | GAATTAATTCCGATATCCATACCTTCATACGAAAACCCTTT |  |
| F-*tsi1* | atttcacacaggaaacagctATGGCCTGTGCTGGTTCTTC | Construction of pBBR-*tsi1* |
| R-*tsi1* | cttggcgtaatcatggtcatTCATTGCACTGGGTCAATATC |  |
| F-*tsi2* | atttcacacaggaaacagct ATGAGACGCTGGTTATTACTAC | Construction of pBBR-*tsi2* |
| R-*tsi2* | cttggcgtaatcatggtcatTCAATAGACCGGATCTGTATC |  |
| F-*tsi3* | atttcacacaggaaacagctATGATAAAGGGTTTTCGTATG | Construction of pBBR-*tsi3* |
| R-*tsi3* | cttggcgtaatcatggtcatTCAGCTGCACTTATCAAGC |  |
| F1-*tse1* | atgaattcccgggagagctCCCTCAATTGGGCGGCGTCTACG | Construction of *Δtse1* |
| R1-*tse1* | ACCGCAATACATTGCTTCATTACACGGCTCCTATCAAATC |  |
| F2-*tse1* | GTGTAATGAAGCAATGTATTGCGGTAG |  |
| R2-*tse1* | tcccaagcttcttctagagAATAGAGCCAGTTTCTGCGATC |  |
| F0-*tse1* | CAAGGCCCCTGGTTACTG |  |
| R0-*tse1* | TAGCAATACCTCATAATAG |  |
| F1-*tse1tsi1* | atgaattcccgggagagctCCCTCAATTGGGCGGCGTCTACG | Construction of *Δtse1Δtsi1* and *Δtse1Δtsi1::tsi1* |
| R1-*tse1tsi1* | ATCCAGCGCATACAAGTCATTACACGGCTCCTATCAAATC |  |
| F2-*tse1tsi1* | CGTGTAATGACTTGTATGCGCTGGATAC |  |
| R2-*tse1tsi1* | tcccaagcttcttctagagCGGTAACGCTGTGCGATC |  |
| F0-*tse1tsi1* | CAAGGCCCCTGGTTACTG |  |
| R0-*tse1tsi1* | ATGCCTAAGCCAATATTATC |  |
| F-*ptsi1* | ACAGGAAACAGTATTCATGGCCTGTGCTGGTTCTTC |  |
| R-*ptsi1* | AGCAGCCTAGGTTAATCATTGCACTGGGTCAATATC |  |
| F1-*tse2* | catgaattcccgggagagctCTGGCCACCATGAGTCAGCAG | Construction of *Δtse2* |
| R1-*tse2* | AACCAGCGTCTCATTTACACGGCTCCTATCAAATC |  |
| F2-*tse2* | ATTTGATAGGAGCCGTGTAAATGAGACGCTGGTTATTACTACCG |  |
| R2-*tse2* | tcccaagcttcttctagagCACCTCCACATACCCGCCGGGGGCGA |  |
| F0-*tse2* | TATACGTCACAAGCAATGCTG |  |
| R0-*tse2* | GCTCCTGATATTGGCTATC |  |
| F1-*tse2tsi2* | catgaattcccgggagagctCTGGCCACCATGAGTCAGCAG | Construction of *Δtse2Δtsi2* and *Δtse2Δtsi2::tsi2* |
| R1-*tse2tsi2* | GCAATCCCAATTACACGGCTCCTATCAAATC |  |
| F2-*tse2tsi2* | TGATAGGAGCCGTGTAATTGGGATTGCGGTCTAGAGG |  |
| R2-*tse2tsi2* | caagcttcttctagagCCCCTTGCTTTGCGGCTTGGGATAACTC |  |
| F0-*tse2tsi2* | TATACGTCACAAGCAATGCTG |  |
| R0-*tse2tsi2* | CAGACTGCTCAGCGGCCTGC |  |
| F-*ptsi2* | atttcacacaggaaacagctATGAGACGCTGGTTATTACTAC |  |
| R-*ptsi2* | tttaacaaaatattaacgcTCAATAGACCGGATCTGTATC |  |
| F1-*tse3* | catgaattcccgggagagctCAACTTCAAATCAAGCTCT | Construction of *Δtse3* |
| R1-*tse3* | CATTATATGATAAAGGGTTTTCGTATG |  |
| F2-*tse3* | ATACGAAAACCCTTTATCATATAATGACCATATCCCCCTC |  |
| R2-*tse3* | atcccaagcttcttctagagTTAAAGAGAATAACGCGAAAC |  |
| F0-*tse3* | ATACAGAGACATCCGCATCA |  |
| R0-*tse3* | GGCGATTGCCACCGAGAAGGCTATG |  |
| F1-*tse3tsi3* | atgaattcccgggagagctTGTATGATGCCCATCCAATAC | Construction of *Δtse3Δtsi3* and *Δtse3Δtsi3::tsi3* |
| R1-*tse3tsi3* | ATGGTCATTATGTTTAACTTATTGTCTTCAT |  |
| F2-*tse3tsi3* | AGACAATAAGTTAAACATAATGACCATATCCCCCTCT |  |
| R2-*tse3tsi3* | cccaagcttcttctagagTTAAAGAGAATAACGCGAAAC |  |
| F0-*tse3tsi3* | ATACAGAGACATCCGCATCA |  |
| R0-*tse3tsi3* | GGCGATTGCCACCGAGAAGGCTATG |  |
| F-*ptsi3* | ttcacacaggaaacagctATGATAAAGGGTTTTCGTATG |  |
| R-*ptsi3* | ttaacaaaatattaacgcTCAGCTGCACTTATCAAGC |  |
| F-M13 | gtaaaacgacggccagt | Validation primers for pBBR |
| R-M13 | gtcatagctgtttcctg |  |
| F-pet25b | GCAGCCAACTCAGCTTCCTT | Validation primers for pET25b |
| R-pet25b | AGAGGATCGAGATCTCGATC |  |
| F-pRE112 | acatagccccactgttcgt | Validation primers for pRE112 |
| R-pRE112 | ggattggctgagacgaaaa |  |

**Table S3. Functional annotation list of genes analyzed using the VFDB database.**

| **Virulence_factor_id** | **vf_name** | **vf_Keyword** |
| --- | --- | --- |
| VF0268 | HitABC | Iron uptake; ABC transporter |
| VF0286 | PhoP | Regulation |
| VF0091 | Alginate | Antiphagocytosis; Serum resistance |
| VF0272 | FbpABC | Iron uptake; ABC transporter |
| VF0274 | Capsule | Antiphagocytosis; Serum resistance |
| VF0463 | BfmRS | Regulation; Two-component system |
| VF0082 | Type IV pili | Adherence; Twitching motility |
| VF0323 | Capsule | Adherence; Phase variation |
| VF0361 | Capsule | Antiphagocytosis |
| VF0072 | ClpC | Stress protein |
